# Dynamic tracing of sugar metabolism reveals the mechanisms of action of synthetic sugar analogs

**DOI:** 10.1093/glycob/cwab106

**Published:** 2021-10-25

**Authors:** Monique van Scherpenzeel, Federica Conte, Christian Büll, Angel Ashikov, Esther Hermans, Anke Willems, Walinka van Tol, Else Kragt, Marek Noga, Ed E Moret, Torben Heise, Jeroen D Langereis, Emiel Rossing, Michael Zimmermann, M Estela Rubio-Gozalbo, Marien I de Jonge, Gosse J Adema, Nicola Zamboni, Thomas Boltje, Dirk J Lefeber

**Affiliations:** Translational Metabolic Laboratory, Radboud University Medical Center, Geert Grooteplein 10, 6525 GA, Nijmegen, The Netherlands; GlycoMScan B.V., Kloosterstraat 9, RE0329, 5349 AB Oss, The Netherlands; Translational Metabolic Laboratory, Radboud University Medical Center, Geert Grooteplein 10, 6525 GA, Nijmegen, The Netherlands; Department of Neurology, Donders Institute for Brain, Cognition and Behavior, Radboud University Medical Center, Geert Grooteplein 10, 6525 GA, Nijmegen, The Netherlands; Department of Radiation Oncology, Radiotherapy & OncoImmunology Laboratory, Radboud Institute for Molecular Life Sciences, Radboud University Medical Center, Geert Grooteplein Zuid 32, Nijmegen, 6525 GA, The Netherlands; Department of Neurology, Donders Institute for Brain, Cognition and Behavior, Radboud University Medical Center, Geert Grooteplein 10, 6525 GA, Nijmegen, The Netherlands; Department of Neurology, Donders Institute for Brain, Cognition and Behavior, Radboud University Medical Center, Geert Grooteplein 10, 6525 GA, Nijmegen, The Netherlands; Department of Neurology, Donders Institute for Brain, Cognition and Behavior, Radboud University Medical Center, Geert Grooteplein 10, 6525 GA, Nijmegen, The Netherlands; Department of Neurology, Donders Institute for Brain, Cognition and Behavior, Radboud University Medical Center, Geert Grooteplein 10, 6525 GA, Nijmegen, The Netherlands; Translational Metabolic Laboratory, Radboud University Medical Center, Geert Grooteplein 10, 6525 GA, Nijmegen, The Netherlands; Translational Metabolic Laboratory, Radboud University Medical Center, Geert Grooteplein 10, 6525 GA, Nijmegen, The Netherlands; Department of Chemical Biology & Drug Discovery, Utrecht Institute for Pharmaceutical Sciences, Utrecht University, Universiteitsweg 99, 3584 CG Utrecht, 6525 AJ, The Netherlands; Cluster for Molecular Chemistry, Institute for Molecules and Materials, Radboud University Nijmegen, Heyendaalseweg 135, 6525 AJ, Nijmegen, The Netherlands; Radboud Center for Infectious Diseases, Section Pediatric Infectious Diseases, Laboratory of Medical Immunology, Radboud Institute for Molecular Life Sciences, Radboud University Medical Center, Geert Grooteplein 10, 6525 GA, Nijmegen, The Netherlands; Cluster for Molecular Chemistry, Institute for Molecules and Materials, Radboud University Nijmegen, Heyendaalseweg 135, 6525 AJ, Nijmegen, The Netherlands; Institute of Molecular Systems Biology, ETH Zurich, 8093 Zurich, Switzerland; Department of Clinical Genetics, department of Pediatrics, Maastricht University Medical Centre, Universiteitssingel 50, P.O. Box 616, Box 16, 6200 MD, Maastricht, The Netherlands; Radboud Center for Infectious Diseases, Section Pediatric Infectious Diseases, Laboratory of Medical Immunology, Radboud Institute for Molecular Life Sciences, Radboud University Medical Center, Geert Grooteplein 10, 6525 GA, Nijmegen, The Netherlands; Department of Radiation Oncology, Radiotherapy & OncoImmunology Laboratory, Radboud Institute for Molecular Life Sciences, Radboud University Medical Center, Geert Grooteplein Zuid 32, Nijmegen, 6525 GA, The Netherlands; Institute of Molecular Systems Biology, ETH Zurich, 8093 Zurich, Switzerland; Cluster for Molecular Chemistry, Institute for Molecules and Materials, Radboud University Nijmegen, Heyendaalseweg 135, 6525 AJ, Nijmegen, The Netherlands; Translational Metabolic Laboratory, Radboud University Medical Center, Geert Grooteplein 10, 6525 GA, Nijmegen, The Netherlands; Department of Neurology, Donders Institute for Brain, Cognition and Behavior, Radboud University Medical Center, Geert Grooteplein 10, 6525 GA, Nijmegen, The Netherlands

**Keywords:** fluoro sialic acid, glycosylation, metabolic oligosaccharide engineering, sugar metabolism, synthetic sugar analog

## Abstract

Synthetic sugar analogs are widely applied in metabolic oligosaccharide engineering (MOE) and as novel drugs to interfere with glycoconjugate biosynthesis. However, mechanistic insights on their exact cellular metabolism over time are mostly lacking. We combined ion-pair ultrahigh performance liquid chromatography–triple quadrupole mass spectrometry mass spectrometry using tributyl- and triethylamine buffers for sensitive analysis of sugar metabolites in cells and organisms and identified low abundant nucleotide sugars, such as UDP-arabinose in human cell lines and CMP-sialic acid (CMP-NeuNAc) in Drosophila. Furthermore, MOE revealed that propargyloxycarbonyl (Poc)-labeled ManNPoc was metabolized to both CMP-NeuNPoc and UDP-GlcNPoc. Finally, time-course analysis of the effect of antitumor compound 3F_ax_-NeuNAc by incubation of B16-F10 melanoma cells with *N*-acetyl-D-[UL-^13^C6]glucosamine revealed full depletion of endogenous ManNAc 6-phosphate and CMP-NeuNAc within 24 h. Thus, dynamic tracing of sugar metabolic pathways provides a general approach to reveal time-dependent insights into the metabolism of synthetic sugars, which is important for the rational design of analogs with optimized effects.

## Introduction

**Fig. 1:**
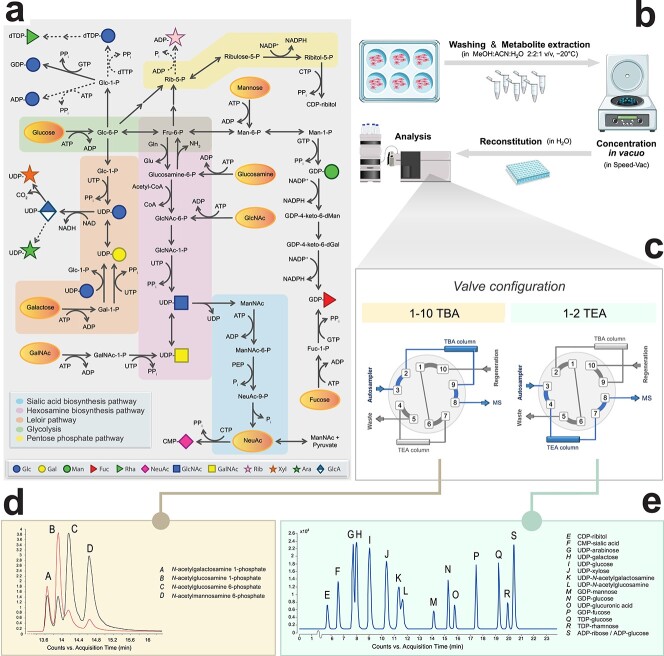
Sugar metabolism as integral part of intracellular metabolism. (A) Overview of the metabolic pathways involved in the generation of nucleotide sugars and their intermediate sugar phosphates in humans. Dashed arrows indicate pathways that remain to be identified in humans. Solid arrows indicate direct reactions, while sequential arrows indicate a series of multiple reactions (details omitted in figure). Yellow ovals indicate where supplemental sugars can enter the metabolic pathways. Abbreviations: Gln, glutamine; Glu, glutamate; PEP, phosphoenolpyruvate; PPi, pyrophosphate; Pi, inorganic phosphate; -P, phosphate; Glc, glucose; Gal, galactose; dGal, deoxygalactose; Fuc, fucose; Fru, fructose; Man, mannose; dMan, deoxymannose; Rib, ribose; NeuNAc, *N*-acetylneuraminic acid; GlcNAc, *N*-acetylglucosamine; GalNAc, *N*-acetylgalactosamine; ManNAc, *N*-acetylmannosamine; AMP/ADP/ATP, adenosine 5'-mono/di/triphosphate; CDP/CTP, cytidine 5'-di/triphosphate; GDP/GTP, guanosine 5'-di/triphosphate; dTDP/dTTP, deoxythymidine 5'-di/triphosphate; UDP/UTP, uridine 5'-di/triphosphate; CoA, coenzyme A; NAD(H), nicotinamide adenine dinucleotide; NADP(H), nicotinamide adenine dinucleotide phosphate. (**B**) Sample preparation consists of six-well cell cultures, followed by extraction of intracellular metabolites for subsequent UHPLC–QqQ analysis. (**C**) The samples can be injected twice via a versatile and automatic switching valve to switch between two different ion pair mobile phases. (**D**) A selection of N-acetylhexosamine phosphate sugars is shown. The red line illustrates the MRM transition 300.0 - > 79.0 m/z, the black line MRM transition 300.0 - > 97.0 m/z. (**E**) Typical chromatogram of 16 nucleotide sugar standards.

Monosaccharides serve as important metabolic intermediates for the production of energy and glycoconjugates across all domains of life. During sugar metabolism, monosaccharides are converted to their corresponding nucleotide sugars in the cellular cytoplasm in a complex metabolic network involving numerous metabolic intermediates and more than 40 currently known enzymes in mammals (Figure 1**A**). Nucleotide sugars serve as building block for glycoproteins, glycolipids and glycosylphosphatidylinositol anchors. These complex glycoconjugates are involved in important biological processes such as cellular communication and signaling and have been associated with numerous human diseases, both acquired (Thanabalasingham et al. 2013; Marotta et al. 2015; Pinho and Reis 2015; Frenkel-Pinter et al. 2017) and genetic (Freeze et al. 2014). Disruptions in sugar metabolism have been linked to various diseases such as cancer (Moriwaki et al. 2009), neurodegenerative (Knight et al. 2014) and infectious (DeVito et al. 2014) disease, some of them being treatable via dietary intervention in sugar metabolism like galactose supplementation in PGM1 myopathy (Tegtmeyer et al. 2014). Its ease of accessibility has made sugar metabolism an attractive target to design synthetic sugar analogs to restore abnormal glycosylation and to study the biological role of glycoconjugates in cells and organisms by metabolic oligosaccharide engineering (MOE).

In MOE, monosaccharides with different chemical groups, such as azide, alkynyl or propargyloxycarbonyl (Poc), enter sugar metabolism via salvage pathways, and are subsequently integrated into glycoconjugates by glycosyltransferases in the endoplasmic reticulum and Golgi apparatus. These chemical groups can then be indirectly visualized after bioorthogonal chemistry by fluorescent detection or mass spectrometry (Dommerholt et al. 2010; Van Wijk et al. 2015; Heise et al. 2017; Woo et al. 2017). Although widely applied, only few studies report on the different substrate specificities of enzymes in sugar metabolism for specific chemical reporter groups. For example, alkyne functionalized GlcNAc (GlcNAlk) was found to be more specific for labeling of O-GlcNAcylated proteins than azide functionalized GlcNAc (GlcNAz) since it was not accepted as substrate by the cytosolic enzyme GALE (Zaro et al. 2011). In addition, specific sialic acid analogs were not accepted as substrate for CMP-sialic acid synthetase (CMAS) (Chinoy et al. 2019).

Synthetic sugars are also developed as inhibitors of sugar metabolic pathways with the aim to develop novel therapeutics (Zhang et al. 2019), such as antimicrobial (Obiol-Pardo et al. 2011; Heise et al. 2018) and anticancer (Büll et al. 2015a; Büll et al. 2016; Ricciardiello et al. 2018) drugs. Similarly, they are used to study the role of glycoconjugates in biological systems. For instance, 6-Diazo-5-oxo-L-norleucine is used to inhibit the synthesis of UDP-GlcNAc. However, being a glutamine analog, it also inhibits other glutamine-dependent enzymes such as glutaminase (Benavides-Rivas et al. 2020). Similarly, 2-deoxyglucose, originally designed as inhibitor of glycolysis, has been shown to exert some of its biological effects by inhibiting protein N-glycosylation via the GDP-mannose pathway (Berthe et al. 2018). Moreover, novel nucleotide sugars have recently been identified in human cells and tissues, including UDP-mannose (Nakajima et al. 2018) and CDP-ribitol (Riemersma et al. 2015), the latter being targeted to develop antimycobacterial drugs (Obiol-Pardo et al. 2011) as it was supposed to be absent in humans. Thus, care should be taken how to interpret the results of studies using synthetic sugars. Together with recent evidence for the existence of additional enzymes in sugar metabolism, the molecular mechanisms and interplay between pathways appear far more complex than anticipated.

To further optimize the effectiveness and specificity of synthetic sugar analogs, detailed knowledge on their metabolism is becoming of key importance. Studies on individual enzymes have provided information on enzyme specificities in different species; however, a more holistic view on cellular metabolism is highly warranted. Analytical methodology has mostly focused on detection of the nucleotide sugar end products by use of triethylamine (TEA) ion-pairing buffer (Räbinä et al. 2001; Turnock and Ferguson 2007; Nakajima et al. 2010; Nakajima et al. 2013). Dedicated methods for analysis of the sugar phosphate intermediates are lacking. Analytical methods for central carbon metabolism, including glycolysis and pentose-phosphate pathway (PPP) are based on chromatographic separation using tributylamine (TBA) ion-pairing buffer (Buescher et al. 2010). Here, we aimed to combine ultrahigh performance liquid chromatography–triple quadrupole mass spectrometry (UHPLC–QqQ) using both TEA and TBA buffers to study the metabolism of synthetic sugars. We first profiled an extended set of nucleotide sugars in 13 commonly used model cell lines and organisms and identified the presence of low abundant nucleotide sugars and found metabolism of the commonly used ManNPoc for MOE toward UDP-GlcNPoc. Lastly, we combined the two ion-pairing methods to investigate the mechanism of action of the potent anti-tumor sugar analog 3F_ax_-NeuNAc. By dynamic tracing of the hexosamine and sialic acid pathways with isotopically labeled [UL-^13^C6]GlcNAc in the presence of 3F_ax_-NeuNAc, we observed full depletion of endogenous *N*-acetyl-mannosamine 6-phosphate (ManNAc-6P) and CMP-NeuNAc within 24 h.

## Results

### UHPLC–QqQ mass spectrometry for analysis of sugar metabolites in model cell lines and organisms

With the aim to establish a single method for analysis of all sugar metabolites (Figure 1**A**), we elaborated on a reversed phase TBA ion-pairing UHPLC–QqQ mass spectrometry method for analysis of polar and anionic metabolites in central carbon metabolism (Buescher et al. 2010). To enable analysis of both nucleotide sugars and sugar-phosphate intermediates, a switching valve was introduced and two similar columns to run batches of TEA and TBA analyses from single samples (Figure 1**B** and **C**).

We selected a set of metabolites representative of glycolysis and the PPP, also in view of their known correlation with sugar metabolism, such as the selective shutdown of the GDP-mannose pathway upon glucose deprivation (Harada et al. 2013) and added pentose- and hexose-phosphates and *N*-acetylhexosamine phosphates by analysis of commercial standards (Figure 1**D**). Nucleotide sugars could be analyzed as well; however, as they eluted at the end of the gradient as broad peaks, separation of isobaric nucleotide sugars was impossible. Therefore, dedicated analysis of nucleotide sugars was performed by separation of 18 nucleotide sugars with 20 mM TEA buffer (Räbinä et al. 2001), resulting in separation of isobaric metabolites, like UDP-glucose and UDP-galactose and (partly) of UDP-GalNAc and UDP-GlcNAc (Figure 1**E**). MRM transitions were obtained via direct infusion of a set of 14 commercially available standards (Table SII) that were also used to optimize ion source settings. Linearity, lower limit of detection (LOD) and lower limit of quantitation (LOQ) were determined as validation in control primary human fibroblasts (Table SII). In brief, low nanomolar concentrations could be detected, the linearity spanned three orders of magnitude with *R*^2^ > 0.99, carry-over was <0.1% for all nucleotide sugars and inter- and intraday coefficients of variation (*n* = 10) were below 10% for all compounds.

**Fig. 2:**
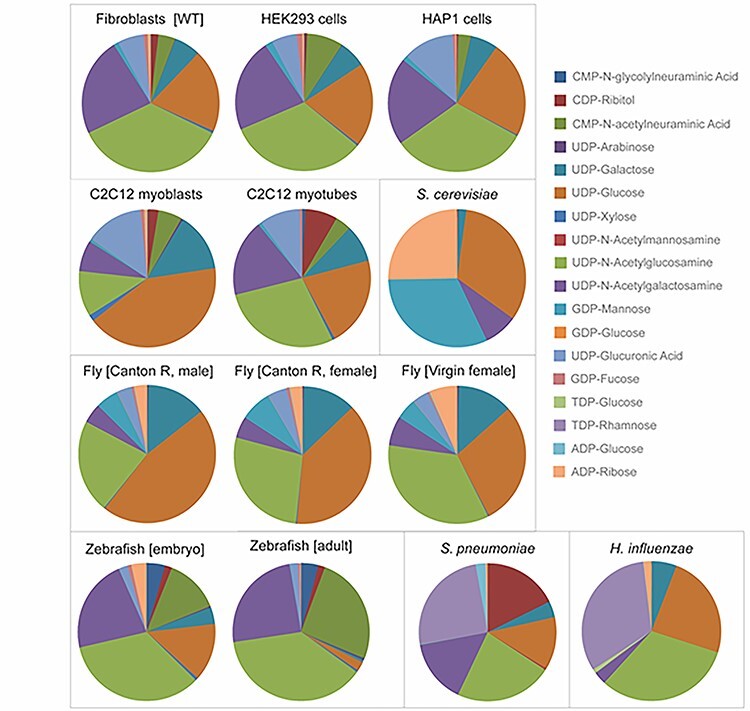
Nucleotide sugar profiles of 13 common model organisms and cell lines. Peak intensities of 18 nucleotide sugars (Table SI) were normalized by the sum of all nucleotide sugars in a given sample and expressed as relative percentage in pie charts. Organisms are shown at the bottom and cell lines at the top. Cell models: human dermal fibroblast (wild-type), human embryonic kidney HEK293 cells, human haploid HAP1 cells, murine C2C12 myoblasts, murine C2C12 myotubes. Model organisms: *Saccharomyces cerevisiae* (yeast), *D. melanogaster* (fly), *D. rerio* (zebrafish), *S. pneumoniae* (gram-positive bacteria), *H. influenzae* (gram-negative bacteria).

As a first step toward diverse applications, we profiled nucleotide sugars in nine commonly studied model cell lines and organisms (Figure 2, Figure S1 and Table SIV). We added UDP-ManNAc and TDP-rhamnose in view of their importance in bacteria and CMP-NeuNGc in view of its importance in mouse cell lines (for conditions, see Table SI). Overall, the three human cell lines showed a similar relative distribution with some differences for example in CMP-NeuNAc and UDP-GlcA. For human HAP1 cells, steady state levels were not significantly influenced by cell culture conditions (Figure S2 and Table SIV). Interestingly, low relative levels of UDP-arabinose were detected (Table SIV, Figure S3), previously identified in plants and in CHO cells (Pabst et al. 2010). The retention time and MRM transitions were identical to commercial UDP-arabinose, which verified its identity (Table SII, Figure S3). For C2C12 mouse myoblasts, a strong change in the relative distribution of nucleotide sugars was observed during differentiation toward myotubes with a strong increase in the levels of CDP-ribitol and UDP-GlcNAc and a relative decrease in UDP-glucose. CDP-ribitol was recently identified in human muscle tissue and is important for the glycosylation of the muscle membrane protein alpha-dystroglycan (van Tol et al. 2019) and might therefore be upregulated during muscle cell differentiation.

Each organism showed its own typical nucleotide sugar profile and could be separated by hierarchical clustering analysis (Figure S4). For example, TDP-rhamnose was detected in *Streptococcus pneumoniae* and *Haemophilus influenzae* and UDP-ManNAc and CDP-ribitol in *H. influenzae*, which are essential nucleotide sugars for synthesis of capsular polysaccharides in these bacteria (Mistou et al. 2016). GDP-mannose levels were most dominant in yeast, as expected because of the high need for mannoprotein synthesis, but were also found at relatively high levels in *Drosophila melanogaster*. In addition, we could identify low relative levels of CMP-NeuNAc in *D. melanogaster*. Although the metabolic pathway for CMP-NeuNAc synthesis has not been broadly investigated in *D. melanogaster*, our finding is in agreement with the presence of sialic acid in *D. melanogaster N*-glycans (Koles et al. 2007) and the intracellular localization of CMP-NeuNAc synthetase and provides a starting point for MOE.

### Metabolic incorporation of Poc-labeled sugars in sialic acid biosynthesis

**Fig. 3:**
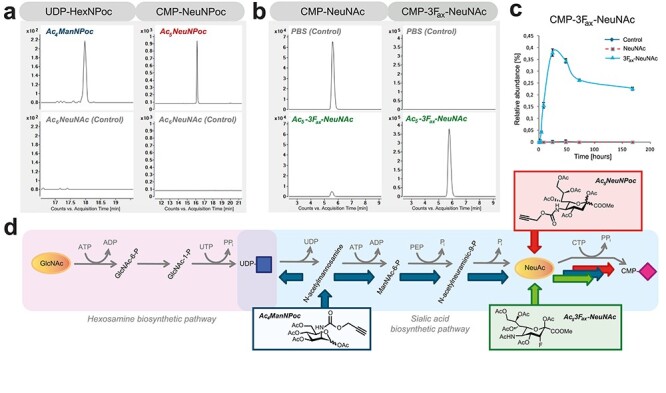
Incorporation of chemical reporter groups in nucleotide sugars. (**A**) Incubation of human dermal fibroblasts for 48 h with 15 μM Ac_4_ManNPoc and Ac_5_NeuNPoc results in metabolic labeling of both UDP-HexNPoc and CMP-NeuNPoc, whereas these nucleotide sugars were absent from 15 μM Ac_5_NeuNAc (controls). (**B**) Formation of CMP-3F_ax_-NeuNAc by feeding B16-F10 cells with Ac_5_-3F_ax_-NeuNAc for 24 h and not by control incubations. (**C**) Time-dependent formation of CMP-3F_ax_-NeuNAc. (**D**) Shown are the de novo CMP-NeuNAc biosynthesis pathway and the salvage pathway of GlcNAc. Indicated in blue arrows is the metabolic route of incorporation of ManNPoc, in red of NeuNPoc and in green of 3F_ax_-NeuNAc.

Over the last decade, MOE has developed as a field to label glycoconjugates by use of synthetic sugars for visualization of cellular glycans or for isolation and subsequent glycomics analysis. A plethora of differently modified monosaccharides is being used for incubation of cells or model organisms with varying labeling efficiencies, possibly related to differences in their metabolism. In previous studies, ManNPoc was shown to be incorporated into glycoconjugates as sialic acid derivative. Its incorporation in cellular glycoconjugates after complete inhibition of sialic acid biosynthesis (Büll et al. 2015b) suggested an additional metabolic fate. To study this, we incubated human fibroblasts with Ac_4_ManNPoc and Ac_5_NeuNPoc and programmed MRM transitions of UDP-GlcNPoc and CMP-NeuNPoc, based on the theoretical mass and fragments of non-derivatized nucleotide sugars (Figure S5**A**, Figure 3**D**, Table SI). After 48 h of incubation with Ac_5_NeuNPoc, the Poc label exclusively appeared in CMP-NeuNPoc, 32% of CMP-NeuNAc. When Ac_4_ManNPoc was administered to cells, 18% labeling was observed in CMP-NeuNPoc, while additionally UDP-HexNPoc was formed for 0.6% as compared with endogenous UDP-GlcNAc (Figure 3**A** and Figure S5**B**). Incubation with Ac_4_ManNPoc or Ac_5_NeuNPoc slightly reduced the levels of endogenous CMP-NeuNAc, whereas the relative levels of other nucleotide sugars remained unaffected (Table SV).

### Dynamic tracing of hexosamine and sialic acid metabolism with *N*-acetyl-D-[UL-^13^C6]glucosamine reveals the cytosolic mechanism of action of sialylation inhibitor 3F_ax_-NeuNAc

**Fig. 4:**
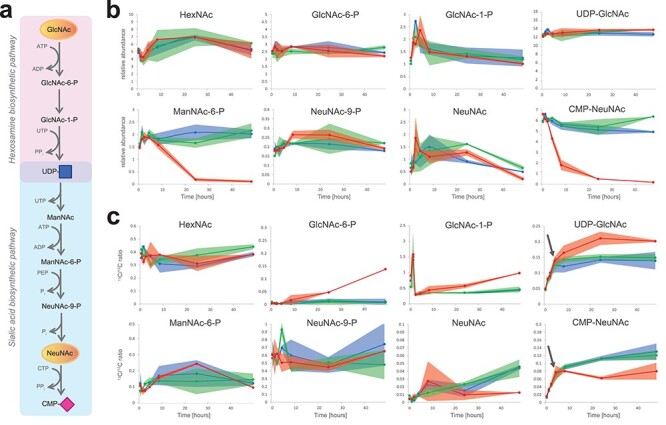
Effect of 3F_ax_-NeuNAc on sialic acid metabolism. (**A**) Position of the presented metabolites in the metabolic pathway. (**B**) B16-F10 mouse myeloma cells were incubated with 100 μM Ac_5_-3F_ax_-NeuNAc for 5 min, 1, 2, 4, 8, 24 and 48 h (red line). Incubations with PBS (blue line) and 100 μM Ac_5_NeuNAc (green line) for the same time points were performed as control. Relative levels of sugar metabolites are presented. (**C**) B16-F10 mouse myeloma cells were incubated with 100 μM Ac_5_-3F_ax_-NeuNAc for the same time points as in (**B**) in the presence of 1 mM [UL-^13^C6]-GlcNAc (red line). Incubations with PBS (blue line) and Ac_5_NeuNAc (green line) for the same time points, both in the presence of 1 mM [UL-^13^C6]-GlcNAc, were performed as control.

As a second chemical glycobiology application to show the importance of analyzing intermediary sugar phosphates, we applied our methodology to study the mechanism of a synthetic inhibitor for sialylation. Global hypersialylation in cancer, as observed for over four decades (Büll et al. 2014; Kohnz et al. 2016), can be inhibited by administration of a fluorinated sialic acid analog, 3F_ax_-NeuNAc (Figure S5**A**), resulting in a reduction in tumor growth in mice (Büll et al. 2013). Its mechanism of action is proposed to be based on dual action on cytosolic CMP-NeuNAc synthesis and the inhibition of Golgi-localized sialyltransferases as confirmed by in vitro enzyme assays (Rillahan et al. 2012). In vivo, neosynthesized CMP-3F_ax_-NeuNAc should be subsequently transported into the Golgi, likely via the CMP-NeuNAc transporter SLC35A1, to inhibit sialyltransferase isoenzymes. To study the metabolism of 3F_ax_-NeuNAc in more detail, we combined our TEA and TBA analytical assays to profile both nucleotide sugars and sugar phosphates intermediates of the hexosamine and sialic acid pathways. We first incubated B16-F10 melanoma cells with peracetylated 3F_ax_-NeuNAc and evaluated its metabolism over time. Following a single pulse, time-dependent formation was observed of CMP-3F_ax_-NeuNAc, with a maximum accumulation at 24 h, which decreased to about 60–70% of maximum levels and remained stably detectable up to at least 7 d (Figure 3**C**). The effect on endogenous metabolites was monitored for 48 h (Figure 4 and Figure S6). A reduction of CMP-NeuNAc appeared after about 4 h, with a further reduction to near depletion after 24 h (Figure 4**B**). In addition, analysis of the intermediate sugar phosphates revealed a marked reduction of ManNAc-6P levels, becoming apparent after 24 h when maximum levels of synthesized CMP-3F_ax_-NeuNAc were reached. No effects on the levels of other endogenous metabolites were observed, nor were the levels of ManNAc-6P influenced by incubations with phosphate-buffered saline (PBS) or peracetylated NeuNAc as controls. The relative levels of other nucleotide sugars were slightly reduced, but this is explained by the high levels of produced CMP-3F_ax_-NeuNAc (Table SVI). These results suggest that in first instance, 3F_ax_-NeuNAc is metabolized to its corresponding CMP derivative, whereas it also competes with endogenous NeuNAc for CMP-NeuNAc synthesis by CMAS, thereby initiating the decrease in CMP-NeuNAc levels. Subsequently, de novo CMP-NeuNAc synthesis by GNE is inhibited by feedback inhibition with CMP-3F_ax_-NeuNAc.

**Fig. 5:**
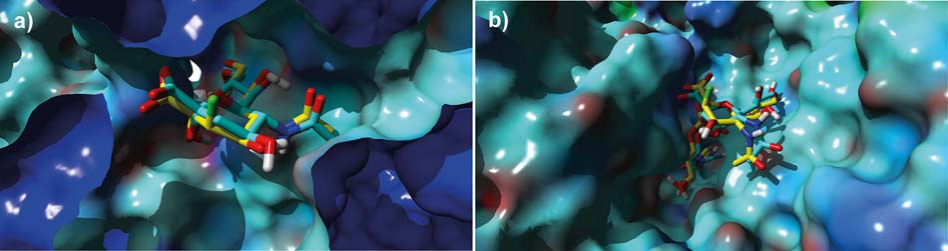
Molecular modeling CMAS and GNE with fluorinated sialic acid analogs. (**A**) Docking of NeuNAc (in blue) and 3F_ax_-NeuNAc (in yellow) in CMAS. (**B**) Docking of CMP-NeuNAc (in blue) and CMP-3F_ax_-NeuNAc (in yellow) in GNE.

We compared the binding of 3F_ax_-NeuNAc and NeuNAc to CMAS and of their CMP derivatives to GNE in silico by molecular modeling. NeuNAc was redocked and 3F_ax_-NeuNAc was docked in the A site of the AB dimer of CMAS (Krapp et al. 2003), using the X-ray structure of murine CMAS as model for the active site of the enzyme. Both molecules overlapped and fitted in the narrow active site (Figure 5**A**). For GNE, CMP-3F_ax_-NeuNAc and CMP-NeuNAc were docked to a human model of GNE (Chen et al. 2016). Both compounds overlapped and bound to the allosteric binding site of GNE (Figure 5 B). Thus, molecular modeling substantiates our findings on the activity of both CMAS and GNE for the fluorinated sialic acid derivatives.

To obtain further insight into the de novo biosynthesis pathway of CMP-NeuNAc in the presence of 3F_ax_-NeuNAc and the involvement of GNE, we performed dynamic tracing of isotopically labeled *N*-acetyl-D-[UL-^13^C6]-glucosamine (^13^C6-GlcNAc) in the presence of Ac_5_-3F_ax_-NeuNAc. Theoretical MRMs for ^13^C6-labeled intermediates of the pathway (Figure 4 A) were programmed and data expressed as ^13^C/^12^C-labeling ratio (Figure 4 C ). In control incubations, ^13^C6-GlcNAc was metabolized to UDP-^13^C6-GlcNAc and CMP-^13^C6-NeuNAc in a time-dependent manner reaching a plateau after about 8 h. In the presence of 3F_ax_-NeuNAc, a relative accumulation of UDP-^13^C6-GlcNAc started to appear between 4 and 8 h, at the same time when metabolism toward CMP-^13^C6-NeuNAc started to decrease. This is suggestive for an inhibition of GNE enzyme activity, as accumulating UDP-GlcNAc is less efficiently converted to ManNAc-6P. More or less in the same time frame, the relative levels of ^13^C6-GlcNAc-6P and ^13^C6-GlcNAc-1P increased as compared with control incubations with PBS and acetylated NeuNAc, indicating a block in the pathway beyond UDP-GlcNAc.

As a complementary line of evidence for the involvement of GNE, we investigated the effect of 3F_ax_-NeuNAc in sialuria fibroblasts (Figure S7), in which the feedback inhibition of GNE by CMP-NeuNAc is deficient. French type sialuria is caused by mutations in the allosteric binding site of GNE, thereby resulting in increased production of NeuNAc and CMP-NeuNAc. Incubation of control fibroblasts with 3F_ax_-NeuNAc resulted in the formation of CMP-3F_ax_-NeuNAc over time and a concomitant decrease in CMP-NeuNAc and also ManNAc-6P, highly similar to the results in B16-F10 melanoma cells. CMP-NeuNAc levels in sialuria fibroblasts were elevated as expected and were reduced slightly upon culturing with 3F_ax_-NeuNAc. Formation of CMP-3F_ax_-NeuNAc was evident but slower as compared with control; however, no effect on ManNAc-6P levels was seen during the incubation time of 48 h. Taken together, our data on ^13^C-GlcNAc tracing together with the data on sialuria fibroblasts are indicative for allosteric inhibition of GNE by CMP-3F_ax_-NeuNAc, which is formed in sufficient amounts after about 4–8 h to affect de novo CMP-NeuNAc synthesis.

Previously, CMP-3F_ax_-NeuNAc was shown to inhibit human sialyltransferase ST6Gal I in in vitro enzyme assays (Rillahan et al. 2012). Interestingly, both the axial and the equatorial variant inhibited ST6Gal I in vitro with similar IC_50_ (IC_50_-values of 9.5 μM for CMP-3F_ax_-NeuNAc and 4.7 μM for CMP-3F_eq_-NeuNAc). However, 3F_eq_-NeuNAc did not show an inhibitory effect on sialylation when incubated with different cell lines, proposed to be caused by the inability of CMAS to metabolize this compound (Kohnz et al. 2016). With the methodology presented here, studies regarding the cellular metabolism of sugar isomers such as 3F_eq_-NeuNAc now become feasible. A relevant question concerns the kinetics of the different inhibitory effects of 3F_ax_-NeuNAc, on cytosolic CMP-NeuNAc and Golgi sialyltransferases. Our data could suggest that the depletion of cytosolic CMP-NeuNAc precedes the direct inhibition of Golgi sialyltransferases. In further studies, it would therefore be interesting to investigate whether CMP-3F_ax_-NeuNAc is itself efficiently transported into the Golgi via SLC35A1 or whether in vivo, 3F_ax_-NeuNAc only acts on cytosolic CMP-NeuNAc levels. Moreover, it would be interesting to combine our methodology for dynamic tracing of sugar metabolic pathways with recent methodology for tracing of ^13^C labeled monosaccharides into glycoconjugates (Wong et al. 2020) to dissect the time-dependent contribution of both inhibitory effects on sialylation.

## Discussion

In summary, we combined analytical methodology for analysis of nucleotide sugars and sugar phosphates to investigate the effects of synthetic sugar analogs on sugar metabolism in a cellular context, at steady state or by dynamic tracing. Separation of isobaric sugar phosphates is challenging and reverse phase chromatography with ion pairing buffers is capable of separating all currently known sugar metabolites. In addition, it allows integrated analysis of additional metabolic pathways in central carbon metabolism that are interlinked with sugar metabolism. Ideally, a single method would be used for analysis of sugar phosphates and nucleotide sugars; however, TBA ion-pairing buffer did not provide separation of isobaric nucleotide sugars. An additional disadvantage is that the use of a dedicated liquid chromatography–mass spectrometry (LC–MS) instrument is preferred in view of the contamination with ion-paring agents. Further developments in ion-mobility mass spectrometry might provide a solution to this problem (King et al. 2019).

Our results from steady-state analysis further expand the repertoire of known human nucleotide sugars. For a long time, synthesis of human glycoconjugates was believed to require nine different nucleotide sugars. Identification of ISPD as disease-causing gene (Roscioli et al. 2012) resulted in the identification of CDP-ribitol in human cells and tissues (Van Tol et al. 2019), whereas more recently, UDP-mannose was detected in human cell lines and mouse tissues (Nakajima et al. 2018). With increased sensitivity, we could detect UDP-arabinose as additional low abundant nucleotide sugar. Possibly, this is formed as byproduct of enzymes that are not 100% specific and form nonclassical metabolites at low rates (Van Schaftingen et al. 2015). The relevance of this nucleotide sugar for glycoconjugate biosynthesis is not clear as it is unknown whether it can indeed be transported into the Golgi apparatus for glycosylation reactions or might interfere with physiological glycosylation processes. The identification of TGDS as disease gene (Ehmke et al. 2014) suggests the presence of even additional nucleotide sugars in human. Thus, this will impact the development of synthetic sugars as antimicrobial drug by targeting sugar metabolic pathways that now also appear to be present in human, such as the ISPD pathway (Obiol-Pardo et al. 2011; Wang et al. 2018).

Another important factor to consider is the fact that intermediary sugar phosphates can influence more distant sugar metabolic pathways. For example, accumulating fructose 1-phosphate inhibits the enzyme mannose phosphate isomerase in the mannose pathway (Jaeken et al. 1996). Since these effects are not easily predictable, care should be taken when designing synthetic compounds for interfering in sugar metabolism and a broad profiling of sugar metabolites and interconnected metabolic pathways as we presented here is recommended to study the specificity and mechanisms of action of synthetic sugar analogs.

In addition to steady state analysis, dynamic tracing of sugar metabolism with isotopically labeled sugars can provide detailed information on the effect of synthetic sugars on individual enzymes in their cellular context. We provide evidence that ManNPoc can be metabolized toward both UDP-GlcNPoc and CMP-NeuNPoc with different efficiencies, which should be considered for MOE studies. It is highly likely that this will depend on the activities of the different enzymes involved, which will differ between cell types and conditions. For instance, we showed that the levels of specific nucleotide sugars change during muscle cell maturation. While tissue-specific regulation of common metabolic pathways such as glycolysis is widely known, this is hardly studied for sugar metabolism. Nonetheless, evidence for tissue-specific regulation is increasing, for example as recently shown for the muscle-specific role of sialic acid catabolism (Wen et al. 2018). Thus, the dynamics and directionality of sugar metabolic pathways should be included in the design of new generation synthetic sugars for precise targeting of glycosylation pathways in a more systematic manner.

In conclusion, we provide methodology to study the interconnected networks of central carbon and sugar metabolism to further optimize the design of synthetic sugars for MOE and therapeutic strategies.

## Experimental section

### Chemicals

Methanol and 2-propanol, both LC–MS grade, the ion pair reagents 3-TBA and TEA-acetic acid, media and sample preparation chemicals as well as all commercial standards (Tables SI and SIII) at the highest available purity were purchased from Sigma-Aldrich (Switzerland), unless stated otherwise. Nanopure water was obtained with an electric resistance of greater than 10 MΩ from a MilliQ purification unit (Millipore, Bedford, MA). TDP-rhamnose was synthesized upon request by CarboSynth (United Kingdom). All studies are in compliance with local ethical rules.

### Extraction of polar metabolites from biological samples

#### Cell lines and incubations with synthetic sugars and sugar isotopes

##### Human dermal fibroblasts (primary) and incubation with Poc sugars

Cells were cultured in six-well plates in M199 medium (PanBioTech), supplemented with 10% fetal calf serum (FCS, Gibco) and 1% penicillin–streptomycin (PenStrep, Gibco). After reaching 70–80% confluence, cells were rinsed twice with 75 mM ammonium carbonate (pH = 7.4) and plates were immediately frozen in liquid nitrogen and stored at −80°C until metabolite extraction. For analysis of the metabolism of Poc-derivatized sugars, ManNPoc and SiaNPoc were synthesized as described before (Büll et al. 2013). Ac_4_ManNPoc, Ac_5_NeuNPoc or Ac_5_NeuAc as control were diluted in culture medium to a final concentration of 15 μM synthetic sugar in the medium and added to growing cells by refreshing the medium. After 48 h incubation with synthetic sugar, cells were rinsed twice with 75 mM ammonium carbonate (pH = 7.4) and plates were immediately frozen in liquid nitrogen and stored at −80°C until metabolite extraction.

##### Human embryonic kidney cells (HEK293)

Cells were cultured on six-well plates in DMEM (Gibco, 4.5 g/L glucose), supplemented with 10% FCS and 1% PenStrep. Upon reaching ~70% confluency, medium was carefully aspirated from the wells and plates immediately placed on ice, and cells were resuspended in 1 mL 75 mM ammonium carbonate pH 7.4, frozen in liquid nitrogen and stored at −80°C until metabolite extraction.

##### Human haploid HAP1 cells and different culture medium compositions

Cells (Horizon Discovery Group, UK) were cultured in six-well plates in IMDM (Gibco, Thermo Fisher Scientific, USA), supplemented with 10% FCS and 1% PenStrep. Cells were passaged 1:10 every 2–3 d. Upon 70–80% confluence, cells were rinsed twice with 75 mM ammonium carbonate (pH = 7.4) and plates were immediately frozen in liquid nitrogen and stored at −80°C until metabolite extraction. To evaluate the influence of glucose and nonessential amino acids in the culture medium on the nucleotide sugar profile, IMDM was replaced with DMEM in various conditions, and cells were cultured for 8 h before harvesting the cells for metabolite extraction. Glucose concentrations used were high (4.5 g/L), low (1 g/L) and 2.75 g/L. Cells were also cultured with and without 1% MEM nonessential amino acids (Gibco).

##### C2C12 mouse myoblasts and differentiation to myotubes

C2C12 myoblasts were cultured in six-well plates on DMEM (Gibco, 4.5 g/L glucose) supplemented with 10% FCS and 1% PenStrep. Upon reaching ~70% confluence, medium was refreshed and after 8 h, the cells were washed and frozen as described for human fibroblasts. For differentiation to myotubes, cells were grown in six-well plates until reaching 100% confluency. Differentiation was initiated by switching the growth medium to DMEM with low concentration of FCS (2%). During differentiation, medium was refreshed every day during 7 d. At 7 d, myotubes were washed twice quickly with 2 mL 75 mM ammonium carbonate pH 7.4 and snap-frozen using liquid nitrogen and stored at −80°C until metabolite extraction.

##### Mouse B16-F10 melanoma cells and dynamic tracing of sialic acid synthesis in the presence of 3F_ax_-NeuNAc

Mouse B16-F10 melanoma cells (ATCC CRL-6475) were cultured in MEM (Gibco) supplemented with 5% fetal bovine serum (FBS; Greiner Bio-one), 1% MEM nonessential amino acids (Gibco), 0.15% sodium bicarbonate (Gibco), 1 mM sodium pyruvate (Gibco), 1.5% MEM vitamins (Gibco) and 1× PenStrep solution (Gibco). The cells were incubated in triplicate for different time points (5 min, 1, 2, 4, 8, 24 and 48 h) with medium containing PBS, 100 μM Ac_5_NeuNAc or 100 μM Ac_5_3F_ax_-NeuNAc, both synthesized as described before (Büll et al. 2013). To correct for the addition of 1 mM [UL-^13^C6]-GlcNAc in the subsequent experiment, 1 mM ^12^C-GlcNAc was added to each of these three conditions. For dynamic tracing of sialic acid biosynthesis in the presence of 100 μM Ac_5_3F_ax_-NeuNAc, cells were incubated in triplicate with 1 mM [UL-^13^C6]-GlcNAc (Omicron biochemicals) in the culture medium in combination with 100 μM Ac_5_3F_ax_-NeuNAc or using 100 μM Ac_5_NeuNAc or PBS as control, as detailed in Figure 4.

##### Human fibroblasts in the presence of 3F_ax_-NeuNAc

Human primary fibroblasts from a control and a patient with French type sialuria due to a mutation in *GNE* in the CMP-NeuNAc binding site were cultured at 37.0°C under 5.0% CO_2_ in culture medium M199, supplemented with 10% FBS and 1% PenStrep. All cultures were tested for mycoplasma infection prior to cultivation. Cells were incubated in triplicate for different time points (0, 4, 8, 24 and 48 h) with growth medium containing PBS or 100 μM Ac_5_3F_ax_-NeuNAc, synthesized as described before (Büll et al. 2013), and metabolites were extracted as described below.

#### Metabolite extraction

All samples were prepared in triplicate and for individual experiments extracted on the same day with identical timings and solvents. Frozen cell pellets as described above were extracted at −20°C with cold 700 μL 2:2:1 (v/v/v) methanol:acetonitrile:water for 2 min. The supernatant was transferred to a separate vial and the extraction repeated with 700 μL cold extraction solvent for 3 min. The two extracts were pooled and centrifuged at 13,000 rpm for 3 min, using a pre-cooled centrifuge (4°C). The resulting supernatants were dried using a vacuum centrifuge (SpeedVac, Thermo Fisher Scientific) at room temperature. The samples were reconstituted in 100 μL MilliQ and centrifuged for 3 min at 14,000 rpm. The supernatant was transferred to polypropylene autosampler vials or 96-well plates for LC–MS analysis, or stored at −80°C until use.

#### Organisms

##### Streptococcus pneumoniae

The gram positive bacterium *S. pneumoniae* serotype 19A was grown on a blood agar plate (BD™ Columbia III Agar with 5% Sheep Blood 254098, containing 12 g/L Pancreatic Digest of Casein, 5 g/L Peptic Digest of Animal Tissue, 3 g/L Yeast Extract, 3 g/L Beef Extract, 1 g/L Corn Starch, 5 g/L Sodium Chloride, 13.5 g/L Agar 4 g/L Growth factors, 5% Sheep Blood, Defibrinated, pH = 7.3 ± 0.2), overnight, in a 37°C, 5% CO_2_ incubator. The next day, single colonies were inoculated into 30 mL THY broth (Difco 249240 and Labconsult SA-NV CON.1702; 3.1 g/L heart, infusion from 500 g, 20 g/L neopeptone, 2 g/L dextrose, 2 g/L sodium chloride, 0.4 g/L disodium phosphate, 2.5 g/L sodium carbonate, 5 g/L yeast) in a 50 mL tube, in a 37°C, 5% CO_2_ incubator and grown until an optical density at a wavelength of 620 nm of 0.3. Then, samples were immediately placed onto ice with NaCl (Merck, 1064041000). In triplicate, 7 mL broth in a 15 mL tube was spun down by centrifugation (1 min, precooled centrifuge at 4°C, 3220 rcf). Supernatant was discarded and the pellet washed with 1.8 mL wash buffer (75 mM ammonium carbonate [Sigma 207861] in MilliQ, buffered with acetic acid [Sigma A6283] at pH 7.4 and cooled to 4°C prior to use). The suspension was transferred to a 2 mL tube and spun down by centrifugation (1 min, precooled centrifuge at 4°C, 25000 rcf). Supernatant was discarded and the pellet was stored at −80°C until metabolite extraction. Metabolites were extracted at −20°C with cold 1 mL 2:2:1 (v/v/v) methanol:acetonitrile:water for 5 min. Then, this was centrifuged at 25000 rcf for 3 min, using a precooled centrifuge at 4°C. The resulting supernatants were dried using a vacuum centrifuge at room temperature and the tubes stored at −80°C until analysis.

##### Haemophilus influenzae

Nontypeable *H. influenzae* strain 86-028NP was grown in 7 mL brain–heart infusion medium (BD biosciences) supplemented with 1 μg/mL hemin and 2 μg/mL ß-nicotinamide adenine dinucleotide (Merck) to an optical density at 620 nm (OD620) of 0.5 in a 50 mL tube. Bacteria were pelleted by centrifugation with 3220 g for 10 min at 4°C. Bacterial pellet was suspended into 2 mL wash buffer (75 mM ammonium carbonate, pH 7.4) and pelleted by centrifugation with 3220 g for 10 min at 4°C. For metabolite extraction, the pellet was suspended into 1 mL 40:40:20 acetonitrile:methanol:water, transferred to a 1.5 mL tube and incubated 5 min at −20°C. Bacteria were pelleted by centrifugation at 16,100 g for 3 min at 4°C. The supernatant was transferred to a new 1.5 mL tube and was dried in a vacuum centrifuge and the tubes stored at −80°C until analysis.

##### Yeast

Fully ^13^C-labeled yeast metabolite extract was a kind gift of Dr. Gerrit Hermann (ISOtopic solutions, Austria) and used for analyses without further purification.

##### Zebrafish

Zebrafish (*Danio rerio*) were housed in recirculating systems on a 14/10 day–night regime. Husbandry was essentially performed as described (Lawrence 2007). Zebrafish embryos (3-d postfertilization; pool of 150 fish; *n* = 3) and juvenile fish (4-week postfertilization; 1 fish/sample; *n* = 4) were euthanized with a lethal dose of tricaine methanesulfonate (MS222). This study was approved by the Animal Ethics Committee of the University of Maastricht (Dier Experimenten Commissie, University of Maastricht) and the Dutch National Central Authority for Scientific Procedures on Animals (Centrale Commissie Dierproeven) (AVD107002016545). Care and use of animals were in agreement with the national and local guidelines. Zebrafish were suspended in 500 μL lysis buffer containing 75 mM ammonium carbonate buffer pH 7.4, homogenized in a potter tube (10 strokes) and subsequently sonicated by the ultrasonic processor UP50H (Hielscher; 2-mm diameter tip, amplitude 175 μm, power density 480 W/cm^2^). Lysates were centrifuged at 11,500 × *g* for 20 min at 4°C. Following protein analysis with the bicinchoninic acid (BCA) Protein Assay Kit, 400 μg of the supernatant were thoroughly mixed with four volumes of cold methanol:acetronitrile (1:1) and incubated on ice for 10 min. After centrifugation at 14,000 *g* for 3 min at 4°C, the supernatant was transferred to a new 1.5 mL tube and was dried in a vacuum centrifuge at room temperature. Tubes were stored at −80°C until analysis.

##### Drosophila melanogaster

Flies were reared at normal fly food at 28°C. After 15 d, adult flies were collected (1–5 d of age), separated on gender and snap-frozen immediately in liquid nitrogen. For each polar metabolite extract, 10 flies were homogenized in 75 mM ammonium carbonate (pH = 7.4) using glass pestles. Protein concentrations were determined using the Bradford protein assay and an equivalent of 200 μg protein was diluted to a total volume of 200 μL using 75 mM ammonium carbonate (pH = 7.4). Polar metabolite extractions were performed by adding four volumes of cold 1:1 methanol:acetonitrile. After 5 min of extraction, samples were centrifuged at 13,000 rpm for 3 min at 4°C. Supernatants were dried using a vacuum centrifuge at room temperature and the tubes stored at −80°C until analysis.

#### Liquid chromatography–mass spectrometry analysis of sugar metabolites

##### UHPLC analysis of nucleotide sugars

Separation of nucleotide sugars was achieved by ion pair-reverse phase chromatography using a modification of previously reported methodology (Buescher et al. 2010). An Agilent 1290 Infinity UHPLC system was used to inject 10 μL of cellular extract samples onto a HSS T3 column (Waters, 2.1 × 150 mm i.d., 1.8-μm particle size) that was maintained at a column temperature of 25°C. Chromatography was performed using a 350 μL/min flow rate and a gradient from 0 to 10% mobile phase B over a 35-min total run time. Mobile phase A1 consisted of 20 mM TEA-acetic acid in H_2_O, and mobile phase B1 consisted of 50% ACN/H_2_O (v/v). The gradient method was as follows (time: % B): 10.0 min: 0% B; 15 min: 4% B; 25 min: 4% B; 26 min: 10% B; 27 min: 10% B; 28 min: 0% B.

##### UHPLC analysis for intracellular metabolome profiling

Separation of a range of polar metabolites swas achieved by an ion pair-reverse phase method as previously reported. An Agilent 1290 Infinity UHPLC system was used to inject 10 μL of metabolite extracts onto a HSS T3 column (Waters, 2.1 × 150 mm i.d., 1.8-μm particle size) that was maintained at a column temperature of 40°C Buescher et al. A gradient of mobile phases A2 (10 mM TBA, 15 mM acetic acid, 5% (v/v) methanol) and B2 (2-propanol) was used to separate the metabolites as indicated Table SIII. The gradient method was as follows (time: % B): 9.5 min: 0% B; 14.5 min: 20% B; 20 min: 45% B; 27 min: 99% B; 31 min: 99% B; 31.5 min: 0% B; 32.0 min: 0% B. The flow rate was 0.25 mL/min, except between 31.5 and 32.0 min, where it was set to 0.15 mL/min.

The two LC methods were operated on the same LC system, by use of separate columns for the individual methods. Analysis of samples using both methods was performed via a switching valve, with switching between methods after each series of samples.

##### QqQ mass spectrometry and MRM acquisition

An Agilent 6490 QqQ LC/MS system with a high-flow iFunnel ionization source, controlled by Agilent’s MassHunter Workstation software (version B.05), was used for all LC-MRM/MS analyses. All acquisition methods used the following parameters: 3500 V capillary voltage and a 2000 V nozzle voltage, a sheath gas flow of 12 L/min (nitrogen) at a temperature of 200°C, a drying gas flow of 15 L/min at a temperature of 200°C, nebulizer gas flow at 20 psi, an MS operating pressure of 5 × 10–5 Torr, and Q1 and Q3 set to unit resolution (0.7 FWHM) and 10 ms dwell time. Agilent Optimizer software was used to find the optimal collision energies and fragment ions for each compound. A default 380 V fragmentor voltage and 4 V cell accelerator potential were used for all MRM ion pairs. The MRM acquisition method was settled with two ion pairs per nucleotide sugar, one quantifier and one qualifier transition (Table SI). Reported responses were based on the quantifier, which was the highest signal producing transition that has been verified to be free of interference. For isotopically labeled nucleotide sugars or synthetically modified nucleotide sugars, the MRM transitions were adapted based on fragmentation knowledge of the nonmodified nucleotide sugars (Table SI). In addition to the 14 commercially available nucleotide sugars (Table SII), CDP-ribitol was synthesized and analyzed as described (Van Tol et al. 2019), CMP-NeuNGc was analyzed based on the conditions for CMP-NeuNAc with calculated mass difference, and UDP-ManNAc was analyzed using the conditions of UDP-GlcNAc. For the intracellular metabolome profiling method, dynamic MRM windows were used as reported in Table SIII.

##### Data analysis

All MRM data were processed using Agilent MassHunter Quantitative Analysis software (Agilent B.07.00) with the Agilent Integrator algorithm for peak integration set with default values. All integrated peaks were manually inspected to ensure correct peak detection and accurate integration. For data interpretation of nucleotide sugars, normalization was performed of peak area over the total peak area of all nucleotide sugars to obtain relative abundances. Differential analyses were performed between conditions and visualized using GraphPad Prism 5.03 or Microsoft Office Excel 2007.

For dynamic tracing of isotopically labeled *N*-acetylglucosamine, the abundance of labeled fraction of each investigated metabolite was calculated using the ratio of ^13^C/^12^C isotopes of a specific metabolite.

### Determination of linearity, LOD and LOQ for nucleotide sugars in fibroblasts

For 14 nucleotide sugars (Table SII) that we detected in human cell lines and for which commercial standards were available, calibration curves were generated to determine the dynamic range by linear regression analysis. A mix of 10 control primary dermal fibroblast extracts was used for the dynamic range determination. Each sample was spiked with an increasing amount of the 14 nucleotide sugars, spanning a 10,000-fold range. The linear range (Table SII) was based on the level of endogenous nucleotide sugars, with the lower standard points designed primarily to determine the LOQ. For determination of the LOD, a signal/noise ratio of 3 was taken. For determination of the LOQ, 10 times a blank injection was performed. The LOQ was calculated by taking the signal of the standards from the blank injection plus 10 times the standard deviation of the noise.

### Structural modeling of CMAS and GNE

For structural modeling of CMAS, PDB file 1qwj (X-ray structure of murine CMAS, Krapp et al. 2003) was used to model the interaction with 3F_ax_-NeuNAc and NeuNAc in the active site of the CMAS enzyme. NeuNAc was redocked and 3F_ax_-NeuNAc was docked in the A site of the AB dimer. The affinities of NeuNAc and 3F_ax_-NeuNAc were predicted to be 50.8 and 49.8 μM, respectively. For structural modeling of GNE, PDB file 4ZHT (Chen et al. 2016) was used to model the interaction of CMP-3F_ax_-NeuNAc and CMP-NeuNAc with the allosteric binding site of human GNE. CMP-3F_ax_-NeuNAc and CMP-NeuNAc bound to the allosteric binding site of GNE with calculated affinities of 2.4 and 0.6 μM, respectively. All modeling was performed with Yasara 17.12.24 (Krieger and Vriend 2014). Local docking with ligand flexibility was performed 25 times with the default Yasara macro dock_runlocal.mcr modified to use the AutoDockLS algorithm.

## Abbreviations

**List of Abbreviations**
*(enzymes, genes and pathways)*
CMAS: cytidine monophosphate N-acetylneuraminic acid synthetase
GALE: UDP-galactose 4-epimerase
GNE: glucosamine (UDP-N-acetyl)-2-epimerase/N-acetylmannosamine kinase
ISPD: isoprenoid synthase domain-containing protein
PGM1: phosphoglucomutase type I
ST6Gal I: ST6 beta-galactoside alpha-2,6-sialyltransferase 1
TGDS: TDP-glucose 4,6-dehydratase
PPP: pentose phosphate pathway
*(suffixes and prefixes)*
[UL-13C6]-: 13C6 labelled isotope (stable)
-Poc: propargyloxycarbonyl (label)
GDP-: guanosine 5’-diphosphate
UDP-: uridine 5’-diphosphate
ADP-: adenosine 5′-diphosphate
CDP-: cytidine 5′-diphosphate
CMP-: cytidine 5′-monophosphate
3Fax-: 3-fluoro (axial)
3Feq-: 3-fluoro (equatorial)
*(sugars and derivatives)*
NeuNAc: N-acetyl-5-neuraminic acid
3Fax-NeuNAc: 3-fluoro (axial)-N-acetyl-5-neuraminic acid
3Feq-NeuNAc: 3-fluoro (equatorial)-N-acetyl-5-neuraminic acid
Ac5NeuNPoc: N-Poc-neuraminic acid
NeuNGc: N-glycolyl-5-neuraminic acid
ManNAc: N-acetyl-D-mannosamine
ManNAc-6P: N-acetyl-D-mannosamine 6-phosphate
Ac4ManNPoc: N-Poc-mannosamine
GlcNAc: N-acetyl-D-glucosamine
GlcNAc-6P: N-acetyl-D-glucosamine 6-phosphate
GlcNAc-1P: N-acetyl-D-glucosamine 1-phosphate
GlcNAlk: alkyne functionalized GlcNAc
GlcNAz: azide functionalized GlcNAc
GalNAc: N-acetyl-D-galactosamine
GlcA: glucuronic acid
*(materials & methods)*
MOE: metabolic oligosaccharide engineering
TEA: triethylamine
TBA: tributylamine
LC-MS: liquid chromatography–mass spectrometry
UHPLC–QqQ: ultra high-performance liquid chromatography coupled with triple quadrupole mass spectrometry
MRM: multiple reaction monitoring
LC-MRM/MS: liquid-chromatography multiple reaction monitoring/mass spectrometry
HSS: high strength silica column
LOQ: limit of quantitation
LOD: limit of detection
FCS: fetal calf serum
IMDM: Iscove′s modified Dulbecco′s medium
DMEM: Dulbecco's modified Eagle's medium
PBS: phosphate-buffered saline
HAP1: human near-haploid cell line
CHO: Chinese hamster ovary cell
THY: Todd-Hewitt broth with yeast extract
OD620: optical density at 620 nm

## Supplementary Material

Sugar_metabolism_supplemental_Figures_cwab106

Supplementary_Table_SI_cwab106

Supplementary_Table_SII_cwab106

Supplementary_Table_SIII_cwab106

Supplementary_Table_SIV_cwab106

Supplementary_Table_SV_cwab106

Supplementary_Table_SVI_cwab106
