# Supplementary material for: Dynamic tracing of sugar metabolism reveals the mechanisms of action of synthetic sugar analogs
Source: Glycobiology. 2021 Oct 25;32(3):239–50. doi: 10.1093/glycob/cwab106 (PMC8966471; doi:10.1093/glycob/cwab106)
Supplement: Sugar_metabolism_supplemental_Figures_cwab106 [file sugar_metabolism_supplemental_figures_cwab106.pdf]

Supporting information

Dynamic analysis of sugar metabolism reveals the mechanisms of action of synthetic sugar analogs

Monique van Scherpenzeel, Federica Conte, Christian Büll, Angel Ashikov, Esther Hermans, Anke Willems, Walinka van Tol, Else Kragt, Ed E. Moret, Torben Heise, Jeroen D. Langereis, Emiel Rossing, Michael Zimmermann, M. Estela Rubio-Gozalbo, Marien I. de Jonge, Gosse J. Adema, Nicola Zamboni, Thomas Boltje and Dirk J. Lefeber*

Supplementary Figure 1: 2

Supplementary Figure 2: 3

Supplementary Figure 3: 4

Supplementary Figure 4: 5

Supplementary Figure 5: 6

Supplementary Figure 6: 7

Supplementary Figure 7: 8

Supplementary Tables 1-6: see separate Files

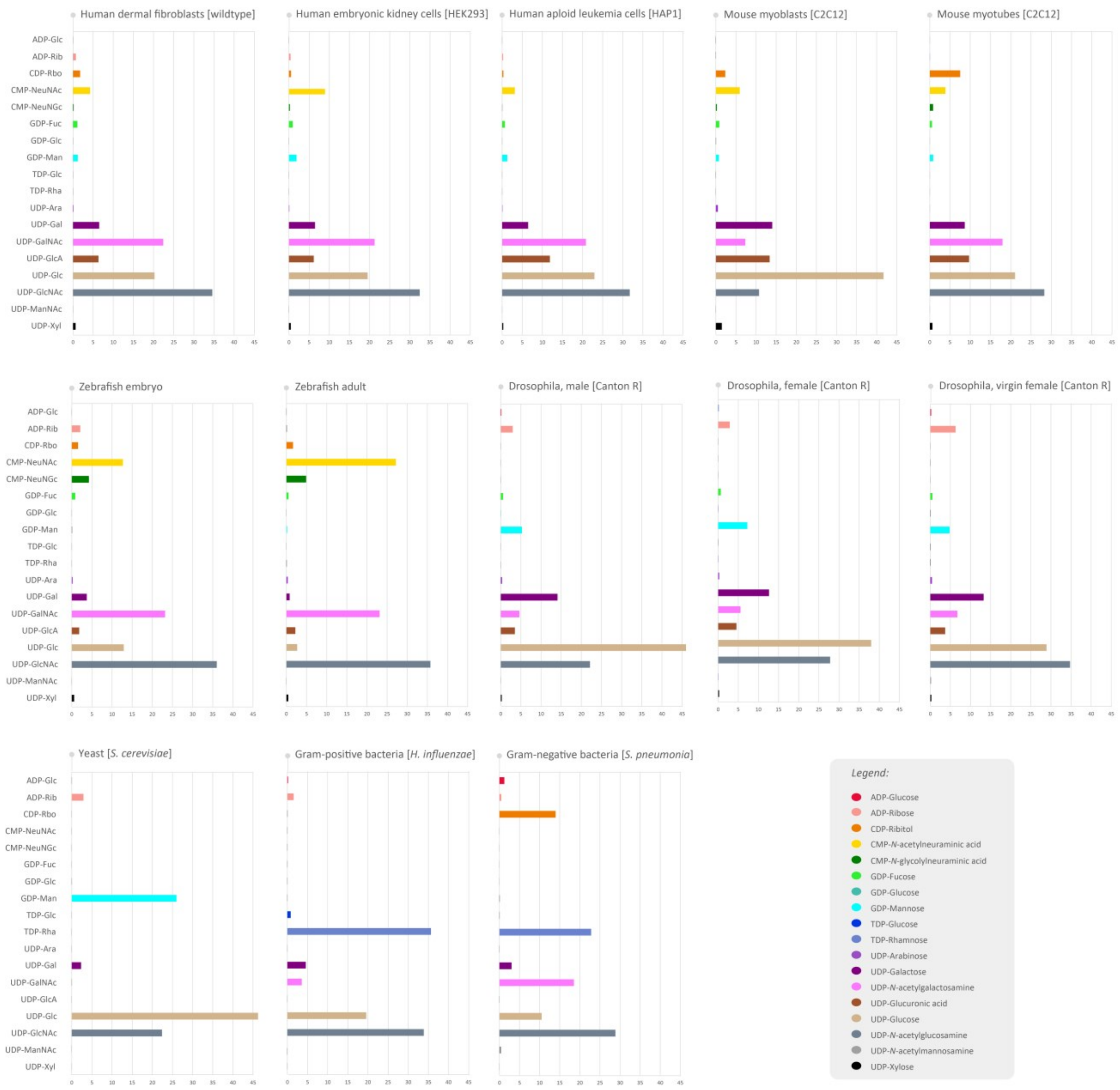

Supplementary Figure 1. Nucleotide sugar profiles of 13 common model organisms and cell lines. Peak intensities of 18 nucleotide sugars (Table SI) were normalized by the sum of all nucleotide sugars in a given sample and expressed as relative percentage in bar charts. Cell models: human dermal fibroblast (wildtype), human embryonic kidney HEK293 cells, human haploid leukemia HAP1 cells, murine C2C12 myoblasts, murine C2C12 myotubes. Model organisms: *Danio rerio* (zebrafish), *Drosophila melanogaster* (fly, strain Canton R), *Saccharomyces cerevisiae* (yeast), *Streptococcus pneumoniae* (gram-positive bacteria), *Haemophilus influenzae* (gram-negative bacteria).

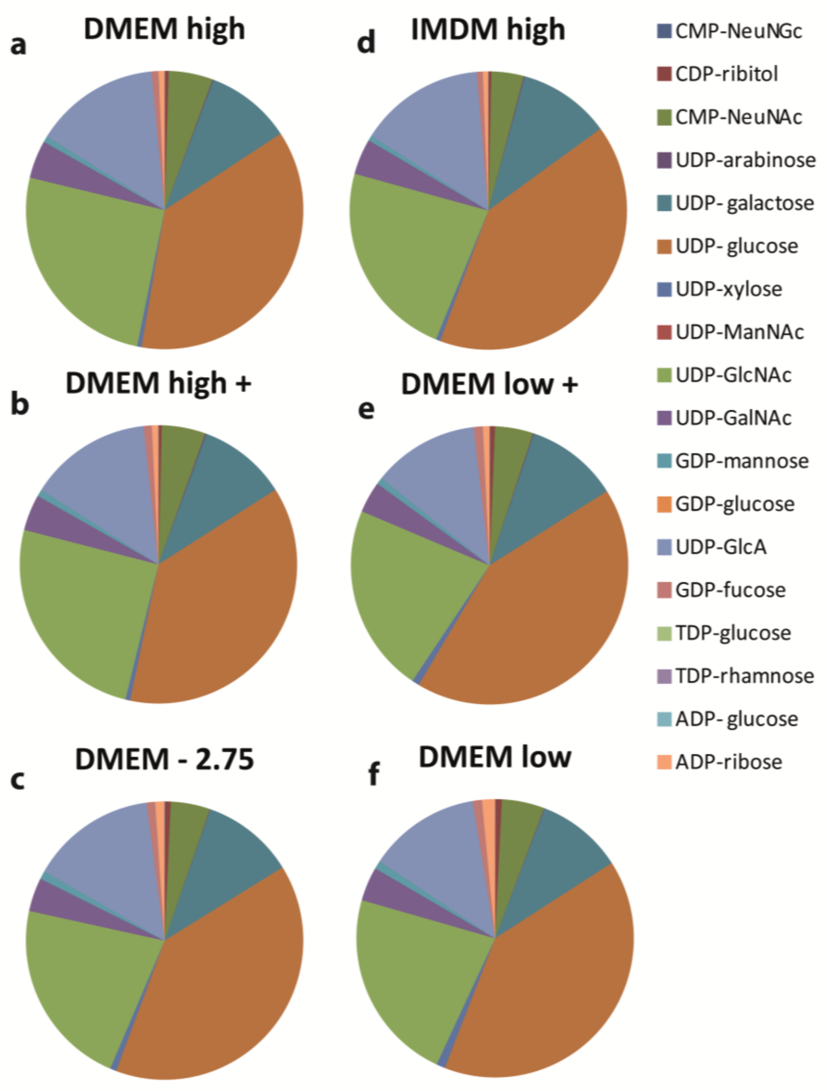

Supplementary Figure 2. The effect of different media compositions on nucleotide sugar profiles in HAP1 cells. Human haploid (HAP1) cells were grown in standard high-glucose IMDM medium, then switched to DMEM with different media compositions and cultured for 8 hours, after which polar metabolites were extracted. Different conditions included glucose concentration (4.5 g/L – high; 1 g/L – low; and 2.75 gr/L) and the presence ('+') or absence of 1% MEM Non-Essential Amino Acids. Experiments were performed in triplo.

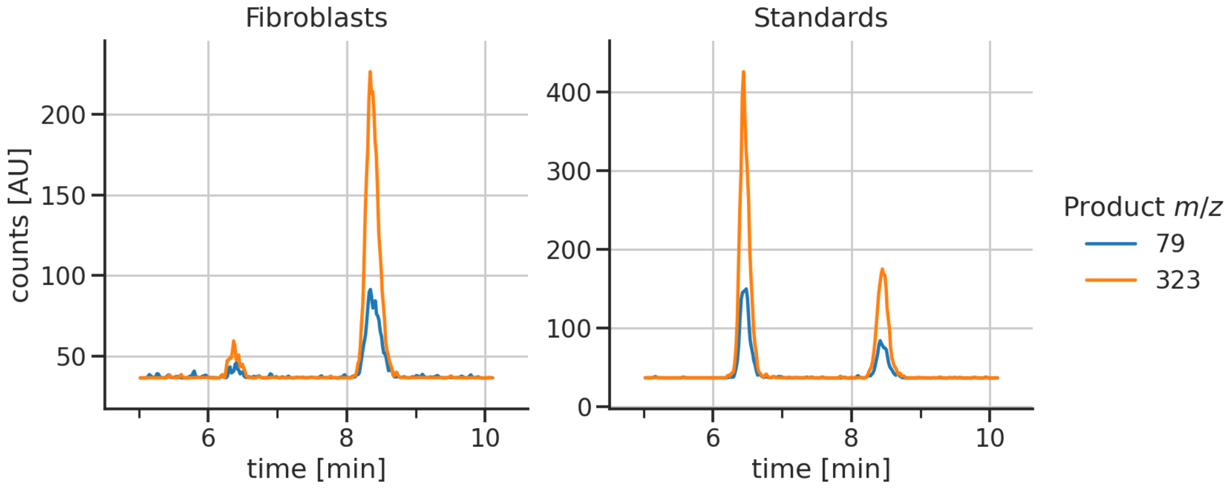

Supplementary Figure 3. UDP-arabinose in human cells. Representative example of the MRM traces of UDP-arabinose (UDP-Ara) and UDP-xylose (UDP-Xyl) in human control fibroblasts. Precursor *m/z* 535, product *m/z* 323 and 79. Left and right panel show traces in fibroblast extracts and chemical standards injected at 400 fmol on column, respectively.

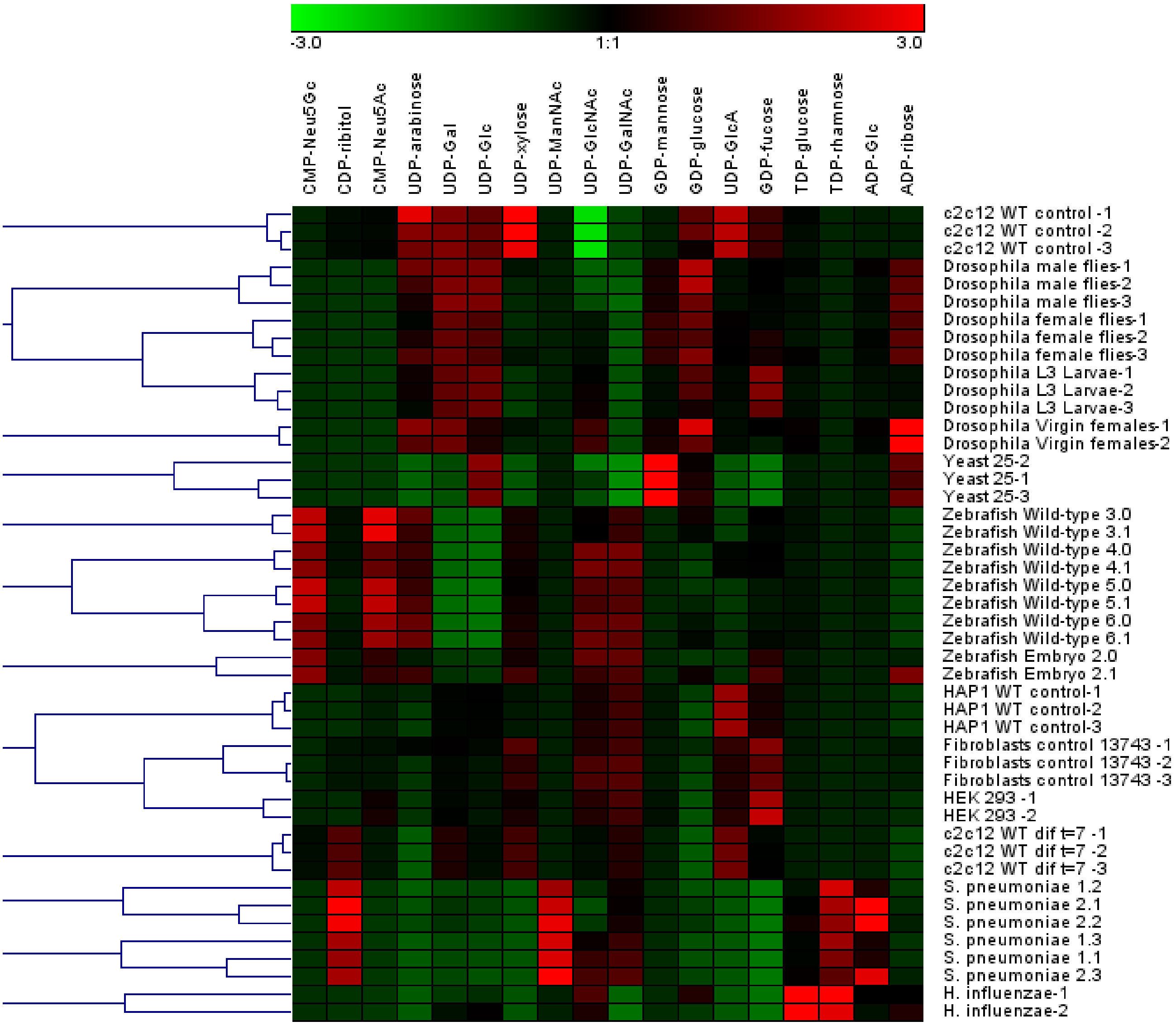

Supplementary Figure 4. Hierarchical clustering of the 9 different model organisms and cell lines. 2D hierarchical clustering (HCL) was performed on the MRM data of in total 46 samples of organisms and cell lines, consisting of the relative abundances of 18 nucleotide sugars. Genesis version 1.7.7 software was used[52] based on average linkage clustering and Pearson correlations.

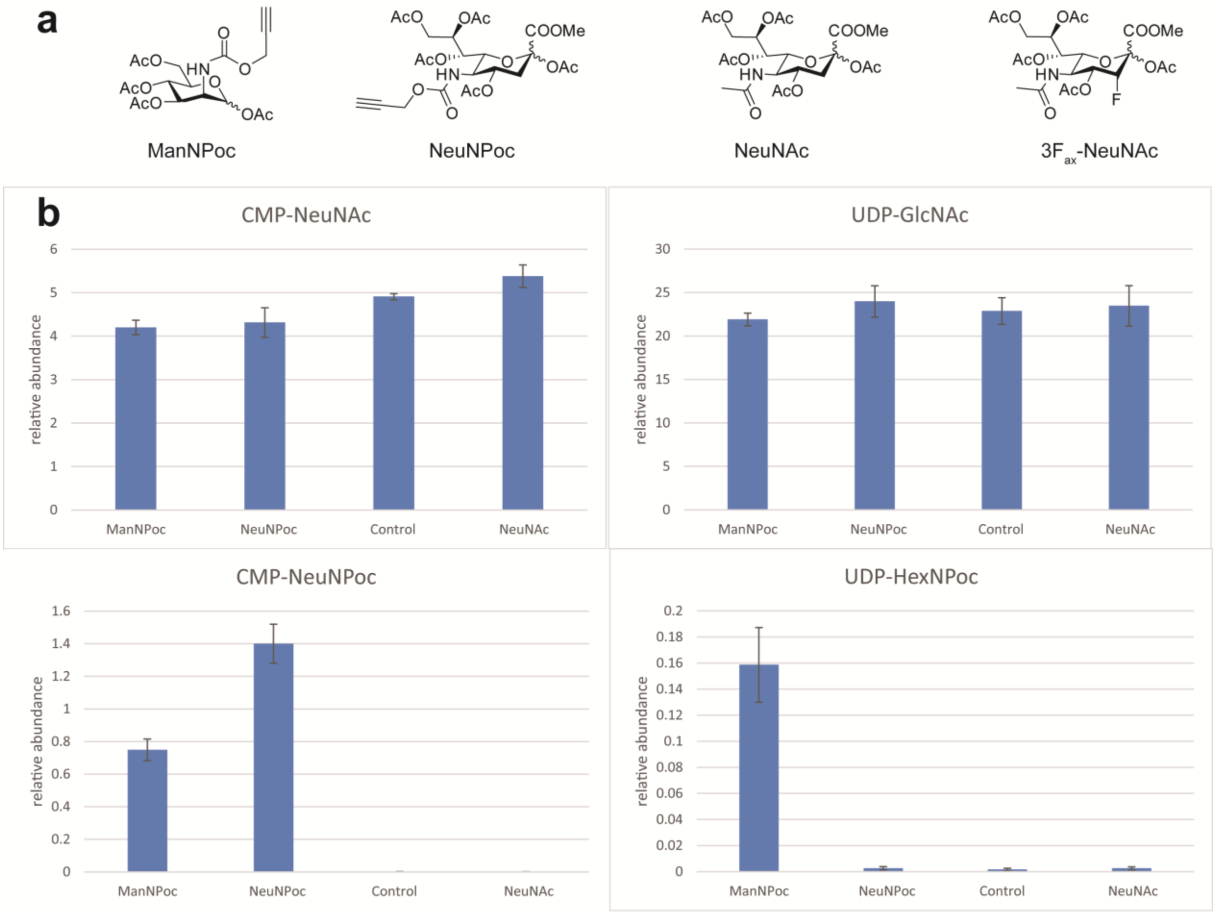

Supplementary Figure 5. Metabolic insights into the uptake of Poc derivatives in fibroblasts. a) Chemical structures of the ManNPoc and NeuNPoc derivatives, sialic acid and its fluorinated derivative as used in this study. b) Relative abundances of CMP-NeuNAc, UDP-GlcNAc, CMP-NeuNPoc and UDP-HexNPoc after 48 hours incubation with respectively 100 µM Ac_5ManNPoc, 100 µM Ac_5NeuNPoc or 100 µM Ac_5NeuNAc. Control fibroblasts incubated with PBS were taken as a control. The average and standard deviation are plotted of triplicate samples.

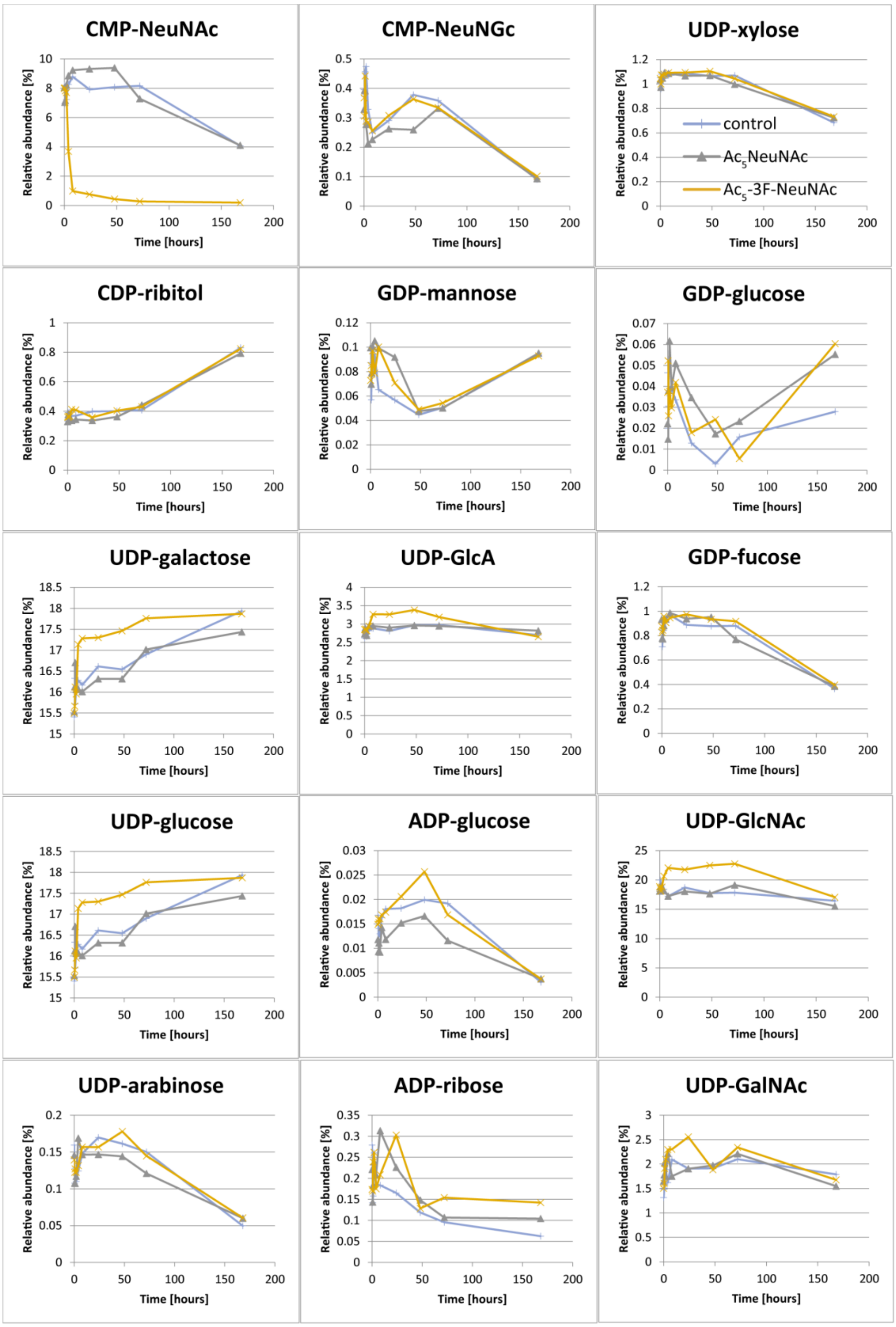

Supplementary Figure 6. Overview of the effect of 3F$_{ax}$-NeuNAc in B16-F10 cells on all nucleotide sugars in time. The relative abundances of nucleotide sugars are shown in time after incubation of B16-F10 cells with Ac$_5$3F$_{ax}$-NeuNAc (orange line), Ac$_5$NeuNAc (green line) or PBS (blue line). Experimental conditions are described in the methods section.

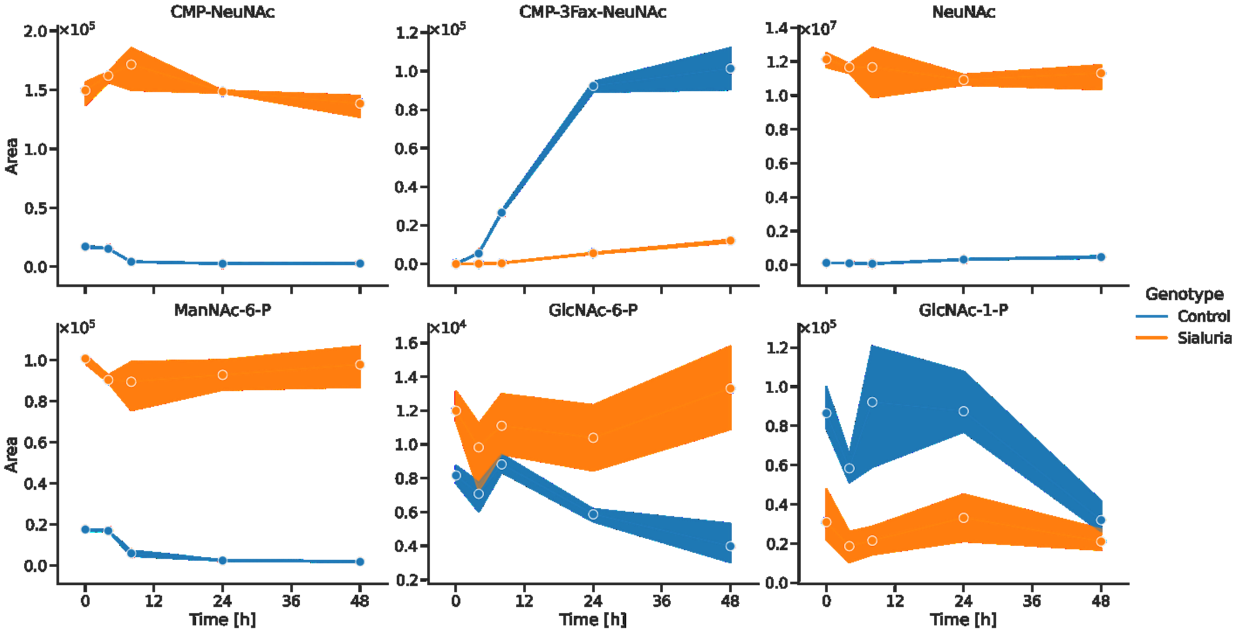

Supplementary Figure 7. Effects of $3F_{ax}$-NeuNAc treatment on intermediates of the sialic acid biosynthesis pathway in fibroblasts. Fibroblast sampled from healthy control (blue) and French type sialuria (orange) were cultured in triplicate and treated with 100 µM $Ac_5 3F_{ax}$-NeuNAc. Plot markers represent mean of three replicates and uncertainty bands represent 95% confidence interval estimated by bootstrapping.
