# Supplementary material for: Dynamic tracing of sugar metabolism reveals the mechanisms of action of synthetic sugar analogs
Source: Glycobiology. 2021 Oct 25;32(3):239–50. doi: 10.1093/glycob/cwab106 (PMC8966471; doi:10.1093/glycob/cwab106)
Supplement: Supplementary_Table_SI_cwab106 [file supplementary_table_si_cwab106.pdf]

Table SI. MRM transitions and collision energies of nucleotide sugars and their isotopically labeled or synthetic analogs.

Compound	Chemical formula	HMDB ID	Retention time (min)	dMRM time window	monoisotopic mass (amu)	Q1 mass (amu)	Q3 mass (amu)	Collision energy (eV)
UDP-xylose	$C_{14}H_{22}N_2O_{16}P_2$	HMDB0001018	9,6	1	536,04	535,03	79,10	30
UDP-xylose	$C_{14}H_{22}N_2O_{16}P_2$	HMDB0001018	9,6	1	536,04	535,03	323,20	20
13C5-UDP-xylose	13C5-$C_9H_{22}N_2O_{16}P_2$		9,6	1	541,04	540,03	79,10	30
13C5-UDP-xylose	13C5-$C_9H_{22}N_2O_{16}P_2$		9,6	1	541,04	540,03	323,20	20
CDP-ribitol	$C_{14}H_{25}N_3O_{15}P_2$	n.a.	4,8	1	537,08	536,07	78,80	50
CDP-ribitol	$C_{14}H_{25}N_3O_{15}P_2$	n.a.	4,8	1	537,08	536,07	322,00	20
13C5-CDP-ribitol	13C5-$C_9H_{25}N_3O_{15}P_2$		4,8	1	542,08	541,07	78,80	50
13C5-CDP-ribitol	13C5-$C_9H_{25}N_3O_{15}P_2$		4,8	1	542,08	541,07	322,00	20
UDP-galactose	$C_{15}H_{24}N_2O_{17}P_2$	HMDB0012305	7,4	1	566,06	565,05	79,00	30
UDP-galactose	$C_{15}H_{24}N_2O_{17}P_2$	HMDB0012305	7,4	1	566,06	565,05	323,10	24
13C6-UDP-galactose	13C6-$C_9H_{24}N_2O_{17}P_2$		7,4	1	572,06	571,05	79,00	30
13C6-UDP-galactose	13C6-$C_9H_{24}N_2O_{17}P_2$		7,4	1	572,06	571,05	323,10	24
UDP-glucose	$C_{15}H_{24}N_2O_{17}P_2$	HMDB0000286	8,3	1	566,06	565,05	79,00	44
UDP-glucose	$C_{15}H_{24}N_2O_{17}P_2$	HMDB0000286	8,3	1	566,06	565,05	323,20	20
13C6-UDP-gluose	13C6-$C_9H_{24}N_2O_{17}P_2$		8,3	1	572,06	571,05	79,00	44
13C6-UDP-glucose	13C6-$C_9H_{24}N_2O_{17}P_2$		8,3	1	572,06	571,05	323,20	20
UDP-GalNAc	$C_{17}H_{27}N_3O_{17}P_2$	HMDB0060522	10,5	1	607,08	606,07	78,90	50
UDP-GalNAc	$C_{17}H_{27}N_3O_{17}P_2$	HMDB0060522	10,5	1	607,08	606,07	273,00	36
13C6-UDP-GalNAc	13C6-$C_{11}H_{27}N_3O_{17}P_2$		10,5	1	613,08	612,07	78,90	50
13C6-UDP-GalNAc	13C6-$C_{11}H_{27}N_3O_{17}P_2$		10,5	1	613,08	612,07	273,00	36
UDP-GlcNAc	$C_{17}H_{27}N_3O_{17}P_2$	HMDB0000290	10,7	1	607,08	606,07	282,10	30
UDP-GlcNAc	$C_{17}H_{27}N_3O_{17}P_2$	HMDB0000290	10,7	1	607,08	606,07	385,10	28
13C6-UDP-GlcNAc	13C6-$C_{11}H_{27}N_3O_{17}P_2$		10,7	1	613,08	612,07	282,10	30
13C6-UDP-GlcNAc	13C6-$C_{11}H_{27}N_3O_{17}P_2$		10,7	1	613,08	612,07	385,10	28
UDP-HexNPoc	$C_{19}H_{27}N_3O_{18}P_2$	n.a.	18,0	1	647,08	646,07	282,10	30
UDP-HexNPoc	$C_{19}H_{27}N_3O_{18}P_2$	n.a.	18,0	1	647,08	646,07	282,10	30
CMP-NeuNAc	$C_{20}H_{31}N_4O_{16}P$	HMDB0001176	6,3	1	614,15	613,14	78,90	54
CMP-NeuNAc	$C_{20}H_{31}N_4O_{16}P$	HMDB0001176	6,3	1	614,15	613,14	321,90	20
13C6-CMP-NeuNAc	13C6-$C_{14}H_{31}N_4O_{16}P$		6,3	1	620,15	619,14	78,90	54
13C6-CMP-NeuNAc	13C6-$C_{14}H_{31}N_4O_{16}P$		6,3	1	620,15	619,14	321,90	20
CMP-3Fax-NeuNAc	$C_{20}H_{30}FN_4O_{16}P$	n.a.	5,8	1	632,13	631,13	78,90	54
CMP-3Fax-NeuNAc	$C_{20}H_{30}FN_4O_{16}P$	n.a.	5,8	1	632,13	631,13	321,90	20
CMP-NeuNPoc	$C_{22}H_{31}N_4O_{17}P$	n.a.	16,2	1	654,13	653,13	78,90	54
CMP-NeuNPoc	$C_{22}H_{31}N_4O_{17}P$	n.a.	16,2	1	654,13	653,13	321,90	20
CMP-NeuNGc	$C_{20}H_{31}N_4O_{17}P$	HMDB0012206	16,2	1	630,14	629,13	78,90	54
CMP-NeuNGc	$C_{20}H_{31}N_4O_{17}P$	HMDB0012206	16,2	1	630,14	629,13	321,90	20
13C6-CMP-NeuNGc	13C6-$C_{14}H_{31}N_4O_{17}P$		16,2	1	636,14	635,13	78,90	54
13C6-CMP-NeuNGc	13C6-$C_{14}H_{31}N_4O_{17}P$		16,2	1	636,14	635,13	321,90	20
GDP-glucose	$C_{16}H_{25}N_5O_{16}P_2$	HMDB0003351	14,9	2	605,08	604,07	362,00	24
GDP-glucose	$C_{16}H_{25}N_5O_{16}P_2$	HMDB0003351	14,9	2	605,08	604,07	78,90	44
13C6-GDP-glucose	13C6-$C_{10}H_{25}N_5O_{16}P_2$		14,9	2	611,08	610,07	362,00	24
13C6-GDP-glucose	13C6-$C_{10}H_{25}N_5O_{16}P_2$		14,9	2	611,08	610,07	78,90	44
GDP-mannose	$C_{16}H_{25}N_5O_{16}P_2$	HMDB0001163	13,6	2	605,08	604,07	424,00	32
GDP-mannose	$C_{16}H_{25}N_5O_{16}P_2$	HMDB0001163	13,6	2	605,08	604,07	78,80	68
13C6-GDP-mannose	13C6-$C_{10}H_{25}N_5O_{16}P_2$		13,6	2	611,08	610,07	78,80	68
13C6-GDP-mannose	13C6-$C_{10}H_{25}N_5O_{16}P_2$		13,6	2	611,08	610,07	424,00	32
GDP-fucose	$C_{16}H_{25}N_5O_{15}P_2$	HMDB0252660	17,1	2	589,08	588,07	442,00	20
GDP-fucose	$C_{16}H_{25}N_5O_{15}P_2$	HMDB0252660	17,1	2	589,08	588,07	424,00	28
GDP-fucose	$C_{16}H_{25}N_5O_{15}P_2$	HMDB0252660	17,1	2	589,08	588,07	78,90	50
13C6-GDP-fucose	13C6-$C_{10}H_{25}N_5O_{15}P_2$		17,1	2	595,08	594,07	442,00	20
13C6-GDP-fucose	13C6-$C_{10}H_{25}N_5O_{15}P_2$		17,1	2	595,08	594,07	424,00	28
13C6-GDP-fucose	13C6-$C_{10}H_{25}N_5O_{15}P_2$		17,1	2	595,08	594,07	78,90	50
ADP-glucose	$C_{16}H_{25}N_5O_{15}P_2$	HMDB0006557	19,9	2	589,08	588,07	346,20	24
ADP-glucose	$C_{16}H_{25}N_5O_{15}P_2$	HMDB0006557	19,9	2	589,08	588,07	241,10	28
13C6-ADP-glucose	13C6-$C_{10}H_{25}N_5O_{15}P_2$		19,9	2	595,08	594,07	346,20	24
13C6-ADP-glucose	13C6-$C_{10}H_{25}N_5O_{15}P_2$		19,9	2	595,08	594,07	241,10	28
UDP-GlcA	$C_{15}H_{22}N_2O_{18}P_2$	HMDB0000935	15,4	2	580,03	579,02	403,00	20
UDP-GlcA	$C_{15}H_{22}N_2O_{18}P_2$	HMDB0000935	15,4	2	580,03	579,02	158,90	42
13C6-UDP-GlcA	13C6-$C_9H_{22}N_2O_{18}P_2$		15,4	2	586,03	585,02	403,00	20
13C6-UDP-GlcA	13C6-$C_9H_{22}N_2O_{18}P_2$		15,4	2	586,03	585,02	158,90	42
ADP-ribose	$C_{15}H_{23}N_5O_{14}P_2$	HMDB0248024	20,0	2	559,07	558,06	346,20	24
ADP-ribose	$C_{15}H_{23}N_5O_{14}P_2$	HMDB0248024	20,0	2	559,07	558,06	78,80	64
13C5-ADP-ribose	13C5-$C_{10}H_{23}N_5O_{14}P_2$		20,0	2	564,07	563,06	346,20	24
13C5-ADP-ribose	13C5-$C_{10}H_{23}N_5O_{14}P_2$		20,0	2	564,07	563,06	78,80	64
dTDP-glucose	$C_{16}H_{26}N_2O_{16}P_2$	HMDB0001328	18,8	2	564,08	563,07	321,00	20
dTDP-glucose	$C_{16}H_{26}N_2O_{16}P_2$	HMDB0001328	18,8	2	564,08	563,07	79,10	48
13C6-dTDP-glucose	13C6-$C_{10}H_{26}N_2O_{16}P_2$		18,8	2	570,08	569,07	321,00	20
13C6-dTDP-glucose	13C6-$C_{10}H_{26}N_2O_{16}P_2$		18,8	2	570,08	569,07	79,10	48
dTDP-rhamnose	$C_{16}H_{26}N_2O_{15}P_2$	HMDB0006354	19,3	2	548,08	547,07	321,00	20
dTDP-rhamnose	$C_{16}H_{26}N_2O_{15}P_2$	HMDB0006354	19,3	2	548,08	547,07	79,10	48
13C6-dTDP-rhamnose	13C6-$C_{10}H_{26}N_2O_{15}P_2$		19,3	2	554,08	553,07	321,00	20
13C6-dTDP-rhamnose	13C6-$C_{10}H_{26}N_2O_{15}P_2$		19,3	2	554,08	553,07	79,10	48
UDP-Arabinose	$C_{14}H_{22}N_2O_{16}P_2$	HMDB0012303	7,1	1	536,04	535,03	322,90	20
UDP-Arabinose	$C_{14}H_{22}N_2O_{16}P_2$	HMDB0012303	7,1	1	536,04	535,03	78,90	30
13C5-UDP-Arabinose	13C5-$C_9H_{22}N_2O_{16}P_2$		7,1	1	541,04	540,03	322,90	20
13C5-UDP-Arabinose	13C5-$C_9H_{22}N_2O_{16}P_2$		7,1	1	541,04	540,03	78,90	30
UDP-ManNAc	$C_{17}H_{27}N_3O_{17}P_2$	HMDB0013112	18,1	1	607,08	606,07	78,90	50
UDP-ManNAc	$C_{17}H_{27}N_3O_{17}P_2$	HMDB0013112	18,1	1	607,08	606,07	273,00	36
