# Supplementary material for: Dynamic tracing of sugar metabolism reveals the mechanisms of action of synthetic sugar analogs
Source: Glycobiology. 2021 Oct 25;32(3):239–50. doi: 10.1093/glycob/cwab106 (PMC8966471; doi:10.1093/glycob/cwab106)
Supplement: Supplementary_Table_SII_cwab106 [file supplementary_table_sii_cwab106.pdf]

Table SII. Acquisition parameters and results of validation for 14 nucleotide sugars.

Compound	HMDB ID	Retention time (min)	Lower limit of detection (µM)	Lower limit of quantification (µM)	Linear range (log3)	Monoisotopic mass (amu)	Q1 mass (amu)	Q3 mass (amu)	Collision energy (eV)	Quantifier/Qualifier
CMP-NeuNAc	HMDB0001176	6,3	0,001	0,003	3 nM - 3,13 µM	614,15	613,10	321,90	20	Quantifier
CMP-NeuNAc	HMDB0001176	6,3	0,001	0,003	3 nM - 3,13 µM	614,15	613,10	78,90	54	Qualifier
UDP-galactose	HMDB0000302	7,4	0,001	0,003	3 nM - 6,25 µM	566,06	565,10	323,10	24	Quantifier
UDP-galactose	HMDB0000302	7,4	0,001	0,003	3 nM - 6,25 µM	566,06	565,10	79,00	30	Qualifier
UDP-glucose	HMDB0000286	8,3	0,001	0,003	3 nM - 6,25 µM	566,06	565,10	323,20	20	Quantifier
UDP-glucose	HMDB0000286	8,3	0,001	0,003	3 nM - 6,25 µM	566,06	565,10	79,00	44	Qualifier
UDP-GalNAc	HMDB0060522	10,5	0,009	0,030	31 nM - 3,13 µM	607,08	606,10	273,00	36	Quantifier
UDP-GalNAc	HMDB0060522	10,5	0,009	0,030	31 nM - 3,13 µM	607,08	606,10	78,90	50	Qualifier
UDP-GlcNAc	HMDB0000290	10,7	0,003	0,009	9 nM - 3,13 µM	607,08	606,10	385,10	28	Quantifier
UDP-GlcNAc	HMDB0000290	10,7	0,003	0,009	9 nM - 3,13 µM	607,08	606,10	282,10	30	Qualifier
GDP-mannose	HMDB0001163	13,6	0,003	0,009	9 nM - 3,13 µM	605,08	604,10	424,00	32	Quantifier
GDP-mannose	HMDB0001163	13,6	0,003	0,009	9 nM - 3,13 µM	605,08	604,10	78,80	68	Qualifier
dTDP-glucose	HMDB0062805	18,8	0,003	0,009	9 nM - 1,25 µM	564,08	563,10	321,00	20	Quantifier
dTDP-glucose	HMDB0062805	18,8	0,003	0,009	9 nM - 1,25 µM	564,08	563,10	79,10	48	Qualifier
GDP-glucose	HMDB0003351	14,9	0,003	0,009	9 nM - 3,13 µM	605,08	604,10	362,00	24	Quantifier
GDP-glucose	HMDB0003351	14,9	0,003	0,009	9 nM - 3,13 µM	605,08	604,10	78,90	44	Qualifier
GDP-fucose	HMDB0252660	17,1	0,003	0,009	9 nM - 1,25 µM	589,08	588,10	442,00	20	Quantifier
GDP-fucose	HMDB0252660	17,1	0,003	0,009	9 nM - 1,25 µM	589,08	588,10	424,00	28	Qualifier
GDP-fucose	HMDB0252660	17,1	0,003	0,009	9 nM - 1,25 µM	589,08	588,10	78,90	50	(extra)
ADP-glucose	HMDB0006557	19,9	0,003	0,009	9 nM - 9,375 µM	589,08	588,10	346,20	24	Quantifier
ADP-glucose	HMDB0006557	19,9	0,003	0,009	9 nM - 9,375 µM	589,08	588,10	241,10	28	Qualifier
ADP-ribose	HMDB0248024	20	0,006	0,020	22 nM - 12,5 µM	559,07	558,10	346,20	24	Quantifier
ADP-ribose	HMDB0248024	20	0,006	0,020	22 nM - 12,5 µM	559,07	558,10	78,80	64	Qualifier
UDP-xylose	HMDB0001018	9,6	0,003	0,009	9 nM - 3,13 µM	536,04	535,00	323,20	20	Quantifier
UDP-xylose	HMDB0001018	9,6	0,003	0,009	9 nM - 3,13 µM	536,04	535,00	79,10	30	Qualifier
UDP-arabinose	HMDB0012303	7,1	0,001	0,003	3 nM - 3,13 µM	536,04	535,00	322,90	20	Quantifier
UDP-arabinose	HMDB0012303	7,1	0,001	0,003	3 nM - 3,13 µM	536,04	535,00	78,90	30	Qualifier
UDP-GlcA	HMDB0000935	15,4	0,001	0,003	3 nM - 9,375 µM	580,03	579,00	403,00	20	Quantifier
UDP-GlcA	HMDB0000935	15,4	0,001	0,003	3 nM - 9,375 µM	580,03	579,00	158,90	42	Qualifier
