# Supplementary material for: Dynamic tracing of sugar metabolism reveals the mechanisms of action of synthetic sugar analogs
Source: Glycobiology. 2021 Oct 25;32(3):239–50. doi: 10.1093/glycob/cwab106 (PMC8966471; doi:10.1093/glycob/cwab106)
Supplement: Supplementary_Table_SIII_cwab106 [file supplementary_table_siii_cwab106.pdf]

**Table SIII. Acquisition parameters of metabolites as analyzed in the TBA method.**

| Compound name | Chemical formula | HMDB ID | Retention time (min) | Monoisotopic mass (amu) | Q1 mass (amu) | Q3 mass (amu) | Collision energy (eV) |
|---|---|---|---|---|---|---|---|
| Acetyl coenzyme A | C23H38N7O17P3S | HMDB0001206 | 24,0 | 809,13 | 808,10 | 408,00 | 36 |
| N-Acetyl-D-galactosamine | C8H15NO6 | HMDB0000212 | 1,5 | 221,09 | 220,10 | 119,00 | 0 |
| N-Acetyl-D-galactosamine 6-phosphate | C8H16NO9P | HMDB0059626 | 14,2 | 301,06 | 300,00 | 97,00 | 16 |
| N-Acetyl-D-galactosamine 6-phosphate | C8H16NO9P | HMDB0059626 | 14,2 | 301,06 | 300,00 | 79,00 | 36 |
| N-Acetyl-D-glucosamine | C8H15NO6 | HMDB0000215 | 1,5 | 221,09 | 220,10 | 59,00 | 20 |
| N-Acetyl-D-glucosamine 1-phosphate | C8H16NO9P | HMDB0001367 | 14,0 | 301,06 | 300,00 | 97,00 | 18 |
| N-Acetyl-D-glucosamine 1-phosphate | C8H16NO9P | HMDB0001367 | 14,0 | 301,06 | 300,00 | 79,00 | 48 |
| N-Acetyl-D-glucosamine 6-phosphate | C8H16NO9P | HMDB0001062 | 13,8 | 301,06 | 300,00 | 97,00 | 16 |
| N-Acetyl-D-glucosamine 6-phosphate | C8H16NO9P | HMDB0001062 | 13,8 | 301,06 | 300,00 | 79,00 | 36 |
| N-Acetyl-D-mannosamine | C8H15NO6 | HMDB0001129 | 1,5 | 221,09 | 220,10 | 59,00 | 0 |
| N-Acetyl-D-mannosamine 6-phosphate | C8H16NO9P | HMDB0062500 | 14,5 | 301,06 | 300,10 | 87,10 | 9 |
| N-Acetyl-D-mannosamine 6-phosphate | C8H16NO9P | HMDB0062500 | 14,5 | 301,06 | 300,10 | 78,90 | 41 |
| N-Acetylneuraminic acid | C11H19NO9 | HMDB0000230 | 7,0 | 309,11 | 308,10 | 169,90 | 10 |
| N-Acetylneuraminic acid | C11H19NO9 | HMDB0000230 | 7,0 | 309,11 | 308,10 | 87,00 | 14 |
| N-Acetylneuraminic acid 9-phosphate | C13H21NO10 | HMDB0000794 | 20,9 | 351,12 | 388,10 | 79,00 | 41 |
| Adenine | C5H5N5 | HMDB0000034 | 2,9 | 135,05 | 134,00 | 107,00 | 18 |
| Adenine | C5H5N5 | HMDB0000034 | 2,9 | 135,05 | 134,00 | 92,10 | 20 |
| Adenosine 5-monophosphate | C10H14N5O7P | HMDB0000045 | 19,0 | 347,06 | 346,00 | 97,00 | 24 |
| Adenosine 5-monophosphate | C10H14N5O7P | HMDB0000045 | 19,0 | 347,06 | 346,00 | 79,00 | 38 |
| Adenosine 5-diphosphate | C10H15N5O10P2 | HMDB0001341 | 22,0 | 427,03 | 426,00 | 328,00 | 16 |
| Adenosine 5-diphosphate | C10H15N5O10P2 | HMDB0001341 | 22,0 | 427,03 | 426,00 | 159,00 | 28 |
| Adenosine 5-triphosphate | C10H16N5O13P3 | HMDB0000538 | 23,2 | 507,00 | 506,00 | 408,10 | 22 |
| Adenosine 5-triphosphate | C10H16N5O13P3 | HMDB0000538 | 23,2 | 507,00 | 506,00 | 159,00 | 38 |
| L-Arabinose | C5H10O5 | HMDB0000646 | 3,0 | 150,05 | 149,00 | 89,10 | 4 |
| L-Arabinose | C5H10O5 | HMDB0000646 | 3,0 | 150,05 | 149,00 | 59,20 | 12 |
| L-Arabitol | C5H12O5 | HMDB0001851 | 1,5 | 152,07 | 151,00 | 89,00 | 12 |
| L-Arabitol | C5H12O5 | HMDB0001851 | 1,5 | 152,07 | 151,00 | 71,02 | 16 |
| CDP-ribitol | C14H25N3O15P2 | n.a. | 20,5 | 537,08 | 536,10 | 322,00 | 20 |
| CDP-ribitol | C14H25N3O15P2 | n.a. | 20,5 | 537,08 | 536,10 | 78,80 | 50 |
| CMP-NeuNAc | C20H31N4O16P | HMDB0001176 | 20,5 | 614,15 | 613,14 | 321,90 | 20 |
| CMP-NeuNAc | C20H31N4O16P | HMDB0001176 | 20,5 | 614,15 | 613,14 | 78,90 | 54 |
| Cytidine | C9H13N3O5 | HMDB0000089 | 1,8 | 243,09 | 242,10 | 109,00 | 8 |
| Cytidine | C9H13N3O5 | HMDB0000089 | 1,8 | 243,09 | 242,10 | 42,20 | 16 |
| Cytidine 5-monophosphate | C9H14N3O8P | HMDB0000095 | 15,5 | 323,05 | 322,00 | 97,00 | 22 |
| Cytidine 5-monophosphate | C9H14N3O8P | HMDB0000095 | 15,5 | 323,05 | 322,00 | 79,00 | 44 |
| Cytidine 5-diphosphate | C9H15N3O11P2 | HMDB0001546 | 21,0 | 403,02 | 402,01 | 158,92 | 24 |
| Cytidine 5-diphosphate | C9H15N3O11P2 | HMDB0001546 | 21,0 | 403,02 | 402,01 | 79,00 | 48 |
| Cytidine 5-triphosphate | C9H16N3O14P3 | HMDB0000082 | 23,0 | 482,98 | 482,00 | 158,80 | 40 |
| Cytidine 5-triphosphate | C9H16N3O14P3 | HMDB0000082 | 23,0 | 482,98 | 482,00 | 79,00 | 40 |
| D-Erythrose 4-phosphate | C4H9O7P | HMDB0001321 | 14,0 | 200,01 | 199,00 | 97,00 | 14 |
| D-Erythrose 4-phosphate | C4H9O7P | HMDB0001321 | 14,0 | 200,01 | 199,00 | 79,00 | 48 |
| D-Fructose 1-phosphate | C6H12O6 | HMDB0062538 | 14,0 | 180,06 | 259,00 | 97,00 | 14 |
| D-Fructose 1-phosphate | C6H12O6 | HMDB0062538 | 14,0 | 180,06 | 259,00 | 79,00 | 48 |
| D-Fructose 6-phosphate | C6H13O9P | HMDB0000124 | 13,5 | 260,03 | 259,00 | 97,00 | 14 |
| D-Fructose 6-phosphate | C6H13O9P | HMDB0000124 | 13,5 | 260,03 | 259,00 | 79,00 | 48 |
| D-Fructose 1,6-bisphosphate | C6H14O12P2 | HMDB0001058 | 20,3 | 340,00 | 338,90 | 241,01 | 12 |
| D-Fructose 1,6-bisphosphate | C6H14O12P2 | HMDB0001058 | 20,3 | 340,00 | 338,90 | 97,00 | 22 |
| Galactitol | C6H14O6 | HMDB0000107 | 1,4 | 182,08 | 181,07 | 101,00 | 13 |
| Galactitol | C6H14O6 | HMDB0000107 | 1,4 | 182,08 | 181,07 | 59,00 | 21 |
| Galactonic acid | C6H12O7 | HMDB0000565 | 4,0 | 196,06 | 195,10 | 129,00 | 11 |
| Galactonic acid | C6H12O7 | HMDB0000565 | 4,0 | 196,06 | 195,10 | 75,10 | 18 |
| D-Galactose 1-phosphate | C6H13O9P | HMDB0000645 | 13,4 | 260,03 | 259,00 | 240,90 | 9 |
| D-Galactose 1-phosphate | C6H13O9P | HMDB0000645 | 13,4 | 260,03 | 259,00 | 79,00 | 28 |
| Glucose | C6H12O6 | HMDB0000122 | 1,4 | 180,06 | 179,10 | 89,10 | 5 |
| Glucose | C6H12O6 | HMDB0000122 | 1,4 | 180,06 | 179,10 | 59,00 | 17 |
| D-Glucose 1-phosphate | C6H13O9P | HMDB0001586 | 13,6 | 260,03 | 259,00 | 240,90 | 9 |
| D-Glucose 1-phosphate | C6H13O9P | HMDB0001586 | 13,6 | 260,03 | 259,00 | 79,00 | 28 |
| D-Glucose 6-phosphate | C6H13O9P | HMDB0001401 | 12,9 | 260,03 | 259,00 | 97,00 | 14 |
| D-Glucose 6-phosphate | C6H13O9P | HMDB0001401 | 12,9 | 260,03 | 259,00 | 79,00 | 48 |
| Glucuronic acid | C6H10O7 | HMDB0000127 | 5,0 | 194,04 | 193,00 | 113,00 | 14 |
| L-Glutamine | C5H10N2O3 | HMDB0000641 | 1,4 | 146,07 | 145,10 | 127,00 | 7 |
| L-Glutamine | C5H10N2O3 | HMDB0000641 | 1,4 | 146,07 | 145,10 | 109,00 | 10 |
| Glyceraldehyde 3-phosphate | C3H7O6P | HMDB0001112 | 14,0 | 170,00 | 169,00 | 97,00 | 4 |
| Glyceraldehyde 3-phosphate | C3H7O6P | HMDB0001112 | 14,0 | 170,00 | 169,00 | 79,00 | 28 |
| N-Glycolylneuraminic acid | C11H19NO10 | HMDB0000833 | 6,0 | 325,10 | 324,09 | 116,00 | 13 |
| N-Glycolylneuraminic acid | C11H19NO10 | HMDB0000833 | 6,0 | 325,10 | 324,09 | 87,10 | 25 |
| Guanine | C5H5N5O | HMDB0000132 | 3,0 | 151,05 | 150,00 | 133,02 | 12 |
| Guanine | C5H5N5O | HMDB0000132 | 3,0 | 151,05 | 150,00 | 66,01 | 36 |
| Guanosine | C10H13N5O5 | HMDB0000133 | 8,8 | 283,09 | 282,10 | 150,00 | 17 |
| Guanosine | C10H13N5O5 | HMDB0000133 | 8,8 | 283,09 | 282,10 | 132,90 | 32 |
| Mannitol | C6H14O6 | HMDB0000765 | 14,0 | 182,08 | 181,10 | 71,00 | 22 |
| D-Mannose | C6H12O6 | HMDB0000169 | 1,4 | 180,06 | 179,10 | 89,00 | 4 |
| D-Mannose | C6H12O6 | HMDB0000169 | 1,4 | 180,06 | 179,10 | 59,20 | 16 |
| D-Mannose 1-phosphate | C6H13O9P | HMDB0006330 | 13,5 | 260,03 | 259,00 | 79,00 | 28 |
| D-Mannose 6-phosphate | C6H13O9P | HMDB0001078 | 13,1 | 260,03 | 259,00 | 97,00 | 14 |
| D-Mannose 6-phosphate | C6H13O9P | HMDB0001078 | 13,1 | 260,03 | 259,00 | 79,00 | 48 |
| Phosphoenolpyruvic acid | C3H5O6P | HMDB0000263 | 21,7 | 167,98 | 167,00 | 79,00 | 12 |
| 6-Phosphogluconic acid | C6H13O10P | HMDB0001316 | 14,0 | 276,02 | 275,00 | 79,00 | 50 |
| 5-Phosphoribosyl 1-pyrophosphate | C5H13O14P3 | HMDB0000280 | 16,0 | 389,95 | 388,90 | 291,00 | 9 |
| 5-Phosphoribosyl 1-pyrophosphate | C5H13O14P3 | HMDB0000280 | 16,0 | 389,95 | 388,90 | 177,00 | 25 |
| 5-Phosphoribosyl 1-pyrophosphate | C5H13O14P3 | HMDB0000280 | 16,0 | 389,95 | 388,90 | 79,00 | 50 |
| 5-Phosphoribosylamine | C5H12NO7P | HMDB0001128 | 1,5 | 229 | 228,00 | 97,00 | 10 |
| 5-Phosphoribosylamine | C5H12NO7P | HMDB0001128 | 1,5 | 229 | 228,00 | 79,00 | 48 |

| | | | | | | | |
|---|---|---|---|---|---|---|---|
| Pyruvic acid | C3H4O3 | HMDB0000243 | 15,1 | 88,02 | 87,00 | 43,20 | 4 |
| Ribitol | C5H12O5 | HMDB0000508 | 1,5 | 152,07 | 151,10 | 89,20 | 9 |
| Ribitol | C5H12O5 | HMDB0000508 | 1,5 | 152,07 | 151,10 | 71,10 | 17 |
| Ribitol 5-phosphate | C5H13O8P | n.a. | 13,5 | 232,03 | 231,00 | 97,00 | 12 |
| Ribitol 5-phosphate | C5H13O8P | n.a. | 13,5 | 232,03 | 231,00 | 79,00 | 50 |
| D-Ribose 5-phosphate | C5H11O8P | HMDB0001548 | 13,5 | 230,02 | 229,00 | 97,00 | 10 |
| D-Ribose 5-phosphate | C5H11O8P | HMDB0001548 | 13,5 | 230,02 | 229,00 | 79,00 | 48 |
| D-Ribose 1-phosphate | C5H11O8P | HMDB0001489 | 15,5 | 230,02 | 229,00 | 97,00 | 10 |
| D-Ribose 1-phosphate | C5H11O8P | HMDB0001489 | 15,5 | 230,02 | 229,00 | 79,00 | 48 |
| Ribulose 5-phosphate | C5H11O8P | HMDB0000618 | 14,5 | 230,02 | 229,01 | 97,00 | 14 |
| Ribulose 5-phosphate | C5H11O8P | HMDB0000618 | 14,5 | 230,02 | 229,01 | 79,00 | 48 |
| D-Sedoheptulose 7-phosphate | C7H15O10P | HMDB0001068 | 13,5 | 290,04 | 289,00 | 96,90 | 18 |
| D-Sedoheptulose 7-phosphate | C7H15O10P | HMDB0001068 | 13,5 | 290,04 | 289,00 | 79,00 | 48 |
| D-Sedoheptulose 1,7-bisphosphate | C7H16O13P2 | HMDB0060274 | 21,2 | 370,01 | 369,00 | 97,00 | 14 |
| D-Sedoheptulose 1,7-bisphosphate | C7H16O13P2 | HMDB0060274 | 21,2 | 370,01 | 369,00 | 79,00 | 48 |
| Sorbitol | C6H14O6 | HMDB0000247 | 1,6 | 182,08 | 181,10 | 101,00 | 13 |
| Sorbitol | C6H14O6 | HMDB0000247 | 1,6 | 182,08 | 181,10 | 59,00 | 21 |
| Trehalose | C12H22O11 | HMDB0000975 | 1,5 | 342,12 | 341,00 | 89,01 | 12 |
| UDP-GlcNAc | C17H27N3O17P2 | HMDB0000290 | 20,8 | 607,08 | 606,07 | 385,10 | 28 |
| UDP-GlcNAc | C17H27N3O17P2 | HMDB0000290 | 20,8 | 607,08 | 606,07 | 282,20 | 30 |
| Uridine | C9H12N2O6 | HMDB0000296 | 4,0 | 244,07 | 243,10 | 200,00 | 6 |
| Uridine | C9H12N2O6 | HMDB0000296 | 4,0 | 244,07 | 243,10 | 110,00 | 12 |
| Xylitol | C5H12O5 | HMDB0002917 | 1,5 | 152,07 | 151,10 | 89,00 | 8 |
| Xylitol | C5H12O5 | HMDB0002917 | 1,5 | 152,07 | 151,10 | 71,00 | 8 |
| D-Xylose | C5H10O5 | HMDB0000098 | 4,0 | 150,05 | 149,05 | 89,02 | 4 |
| D-Xylose | C5H10O5 | HMDB0000098 | 4,0 | 150,05 | 149,05 | 71,01 | 4 |
| D-Xylulose 5-phosphate | C5H11O8P | HMDB0000868 | 14,3 | 230,02 | 229,00 | 138,98 | 8 |
| D-Xylulose 5-phosphate | C5H11O8P | HMDB0000868 | 14,3 | 230,02 | 229,00 | 79,00 | 36 |
