# Supplementary material for: Dynamic tracing of sugar metabolism reveals the mechanisms of action of synthetic sugar analogs
Source: Glycobiology. 2021 Oct 25;32(3):239–50. doi: 10.1093/glycob/cwab106 (PMC8966471; doi:10.1093/glycob/cwab106)
Supplement: Supplementary_Table_SIV_cwab106 [file supplementary_table_siv_cwab106.pdf]

Table SIV. Comparative analysis of the relative abundance of nucleotide sugars in 13 organisms or cell lines.

Relative levels of nucleotide sugars in HAP1 cells cultured under different conditions (supplementary Figure 1)

	CMP-NeuNGc	CDP-ribitol	CMP-NeuNAc	UDP-arabinose	UDP-galactose	UDP-glucose	UDP-xylose	UDP-ManNAc	UDP-GlcNAc	UDP-GalNAc	GDP-mannose	GDP-glucose	UDP-GlcA	GDP-fucose	TDP-glucose	TDP-rhamnose	ADP-glucose	ADP-ribose
t=8 DMEM high	0,09	0,39	5,16	0,12	10,01	36,91	0,51	n.d.	25,56	4,40	0,74	n.d.	14,60	0,80	n.d.	n.d.	n.d.	0,70
t=8 IMDM high	0,07	0,30	3,78	0,12	10,75	40,65	0,50	n.d.	23,11	4,15	0,61	n.d.	14,64	0,68	n.d.	n.d.	n.d.	0,63
t=8 DMEM high+	0,08	0,31	5,11	0,15	10,34	37,29	0,55	n.d.	25,17	4,19	0,81	n.d.	14,28	0,95	n.d.	n.d.	n.d.	0,75
t=8 DMEM low+	0,10	0,54	4,47	0,12	10,86	42,47	0,85	n.d.	21,95	3,64	0,81	n.d.	12,37	1,06	n.d.	n.d.	n.d.	0,76
t=8 DMEM - 2,75	0,11	0,61	4,60	0,06	10,80	39,49	0,74	n.d.	22,01	3,91	0,98	n.d.	14,62	0,97	n.d.	n.d.	n.d.	1,07
t=8 DMEM low	0,07	0,71	4,98	0,08	10,07	40,01	1,02	n.d.	22,49	3,95	0,97	n.d.	13,07	1,05	n.d.	n.d.	n.d.	1,51

Relative levels of nucleotide sugars in organisms and cells (Figure 2)

	CMP-NeuNGc	CDP-ribitol	CMP-NeuNAc	UDP-arabinose	UDP-galactose	UDP-glucose	UDP-xylose	UDP-ManNAc	UDP-GlcNAc	UDP-GalNAc	GDP-mannose	GDP-glucose	UDP-GlcA	GDP-fucose	TDP-glucose	TDP-rhamnose	ADP-glucose	ADP-ribose
Human WT fibroblasts	0,16	1,76	4,23	0,12	6,50	20,25	0,65	0,00	34,58	22,41	1,18	0,00	6,32	1,09	0,00	0,00	0,00	0,73
HEK293	0,23	0,51	9,11	0,09	6,47	19,53	0,47	0,00	32,46	21,26	1,86	0,00	6,15	1,45	0,00	0,00	0,00	0,40
HAP1	0,04	0,32	3,14	0,09	6,47	22,95	0,28	0,00	31,74	20,85	1,27	0,00	11,87	0,70	0,00	0,00	0,00	0,25
Mouse C2C12 myoblasts	0,25	2,34	5,94	0,50	14,02	41,69	1,47	0,00	10,73	7,31	0,75	0,00	13,38	0,86	0,00	0,00	0,00	0,64
Mouse C2C12 myotubes	0,82	7,52	3,87	0,02	8,64	21,06	0,67	0,00	28,27	17,96	0,84	0,00	9,71	0,56	0,00	0,00	0,00	0,04
Zebrafish adult	4,89	1,63	27,16	0,34	0,80	2,67	0,47	0,00	35,73	23,14	0,17	0,02	2,22	0,53	0,02	0,08	0,04	0,09
Zebrafish embryo	4,25	1,55	12,70	0,22	3,72	12,90	0,58	0,00	35,99	23,17	0,08	0,02	1,83	0,86	0,01	0,00	0,02	2,11
Canton R male flies	0,00	0,00	0,03	0,32	14,08	45,98	0,24	0,00	22,13	4,64	5,26	0,08	3,49	0,57	0,04	0,00	0,17	2,97
Canton R female flies	0,00	0,01	0,03	0,26	12,62	37,98	0,24	0,00	27,79	5,55	7,24	0,06	4,53	0,65	0,04	0,00	0,15	2,86
Canton R Virgin females	0,00	0,01	0,03	0,41	13,25	28,90	0,27	0,00	34,73	6,73	4,80	0,08	3,71	0,51	0,07	0,00	0,21	6,28
Yeast	0,00	0,01	0,00	0,00	2,31	46,24	0,00	0,00	22,41	0,04	25,98	0,04	0,02	0,00	0,01	0,00	0,04	2,91
Bacteria gram positive	0,02	0,05	0,04	0,01	4,58	19,57	0,02	0,00	33,85	3,58	0,03	0,02	0,01	0,03	0,83	35,63	0,16	1,57
Bacteria gram negative	0,00	13,97	0,00	0,02	3,02	10,51	0,01	0,41	28,85	18,54	0,07	0,01	0,03	0,00	0,06	22,81	1,22	0,46
