# Supplementary material for: Dynamic tracing of sugar metabolism reveals the mechanisms of action of synthetic sugar analogs
Source: Glycobiology. 2021 Oct 25;32(3):239–50. doi: 10.1093/glycob/cwab106 (PMC8966471; doi:10.1093/glycob/cwab106)
Supplement: Supplementary_Table_SV_cwab106 [file supplementary_table_sv_cwab106.pdf]

Table SV. Overview of the effect of ManNPoc and SiaNPoc labeling in fibroblasts on all nucleotide sugars and incorporation of the Poc group.

The relative abundances of nucleotide sugars are shown after incubation of fibroblasts with Ac5ManNPoc, Ac5NeuNPoc, Ac5NeuNAc or PBS. In addition, the incorporation of ManNPoc and SiaNPoc in nucleotide sugars is shown as ratio of the theoretically produced Poc derivatized nucleotide-sugar versus endogenous, non-modified nucleotide sugars.

Sample	CDP-ribitol	CMP-NeuNAc	UDP-galactose	UDP-glucose	UDP-arabinose	UDP-xylose	UDP-GalNAc	UDP-GlcNAc	GDP-glucose	GDP-mannose	UDP-GlcA	CMP-NeuNPoc	GDP-fucose	UDP-HexNPoc	TDP-glucose	ADP-glucose	ADP-ribose
**Average**																	
ManNPoc	0,75	4,20	11,24	40,16	2,14	2,25	3,67	21,90	0,00	0,33	9,40	0,75	1,44	0,16	0,00	0,00	0,90
NeuNPoc	0,75	4,32	11,38	36,50	1,99	2,08	4,58	23,97	0,00	0,32	9,83	1,40	1,34	0,00	0,00	0,00	0,85
NeuNAc	0,77	5,38	13,10	38,10	1,49	1,56	3,81	23,47	0,00	0,33	9,87	0,00	1,22	0,00	0,00	0,00	0,45
Control (PBS)	0,78	4,91	11,83	38,22	1,77	1,86	4,29	22,88	0,00	0,37	10,19	0,00	1,24	0,00	0,00	0,00	0,71
**Percentage Poc labeling**																	
ManNPoc	n.d	18	n.d	n.d	n.d	n.d	n.d	0,6	n.d	n.d	n.d	n.d	n.d	n.d	n.d	n.d	n.d
NeuNPoc	n.d	32	n.d	n.d	n.d	n.d	n.d	n.d	n.d	n.d	n.d	n.d	n.d	n.d	n.d	n.d	n.d
NeuNAc	n.d	n.d	n.d	n.d	n.d	n.d	n.d	n.d	n.d	n.d	n.d	n.d	n.d	n.d	n.d	n.d	n.d
