# Supplementary material for: Dynamic tracing of sugar metabolism reveals the mechanisms of action of synthetic sugar analogs
Source: Glycobiology. 2021 Oct 25;32(3):239–50. doi: 10.1093/glycob/cwab106 (PMC8966471; doi:10.1093/glycob/cwab106)
Supplement: Supplementary_Table_SVI_cwab106 [file supplementary_table_svi_cwab106.pdf]

Table SVI. Overview of the effects of 3Fax-NeuNAc on all nucleotide sugars

A. The relative abundances of nucleotide sugars are shown (in %) after 48 h incubation of B16-F10 cells with Ac_5-3Fax-NeuNAc, Ac_5NeuNAc or PBS (control). In addition, the incorporation of Ac_5-3Fax-NeuNAc is shown as ratio of the theoretically produced fluoro derivatives versus endogenous, non-modified nucleotide sugars.

Sample	CDP-ribitol	CMP-NeuNAc	CMP-3Fax-NeuNAc	UDP-galactose	UDP-glucose	UDP-arabinose	UDP-xylose	UDP-GlcNAc
**Average**								
3Fax-NeuNAc	0,231	0,291	34,474	11,153	33,117	0,086	0,680	1,167
NeuNAc	0,331	9,564	0,006	15,937	48,493	0,124	1,040	1,407
Control (PBS)	0,367	8,278	0,034	16,096	49,014	0,122	1,037	1,519

Sample	UDP-GalNAc	GDP-glucose	GDP-mannose	UDP-GlcA	GDP-fucose	ADP-glucose	TDP-glucose	ADP-ribose
**Average**								
3Fax-NeuNAc	15,939	0,000	0,033	2,068	0,618	0,014	0,001	0,101
NeuNAc	19,111	0,001	0,051	2,790	0,932	0,014	0,001	0,153
Control (PBS)	19,640	0,001	0,049	2,738	0,895	0,016	0,006	0,152

B. The relative abundances of nucleotide sugars are shown (in %) after 0,4, 8, 24 and 48 h of incubation of fibroblast control and Sialuria cells with Ac_5-3Fax-NeuNAc. In addition, the incorporation of Ac_5-3Fax-NeuNAc is shown as ratio of the theoretically produced fluoro derivatives versus endogenous, non-modified nucleotide sugars.

Genotype	Time [h]	CDP-ribitol	CMP-NeuNAc	CMP-3Fax-NeuNAc	UDP-galactose	UDP-glucose	UDP-arabinose	UDP-xylose	UDP-HexNAc	GDP-mannose	UDP-GlcA	GDP-fucose	ADP-ribose
**Control**	**0**	0,42	3,59	0,00	10,35	34,63	0,07	0,83	34,40	0,97	12,05	1,55	1,14
	**4**	0,40	2,69	0,95	9,95	33,91	0,05	0,64	31,94	0,87	16,76	0,93	0,90
	**8**	0,38	0,65	4,03	10,37	35,73	0,04	0,70	31,41	0,81	13,81	1,22	0,81
	**24**	0,39	0,42	14,30	8,90	30,62	0,04	0,61	25,99	0,92	15,58	1,36	0,85
	**48**	0,32	0,46	16,66	8,44	28,04	0,05	0,62	31,16	0,72	11,39	1,37	0,75
**Sialuria**	**0**	0,46	34,50	0,00	6,74	23,34	0,04	0,49	24,10	0,78	7,22	1,03	1,28
	**4**	0,44	34,72	0,02	6,64	22,64	0,03	0,47	23,62	0,74	8,66	0,98	1,01
	**8**	0,44	33,11	0,08	7,09	24,55	0,02	0,46	23,20	0,76	8,26	1,00	1,00
	**24**	0,52	31,33	1,16	7,35	24,99	0,03	0,51	23,33	0,79	7,62	1,06	1,29
	**48**	0,39	31,14	2,72	6,94	23,12	0,04	0,51	24,87	0,73	7,33	1,07	1,11
